# Inhibition of CXXC5 function reverses obesity‐related metabolic diseases

**DOI:** 10.1002/ctm2.742

**Published:** 2022-04-05

**Authors:** Seol Hwa Seo, Eunhwan Kim, Soung‐Hoon Lee, Yong‐ho Lee, Dai Hoon Han, Hyesun Go, Je Kyung Seong, Kang‐Yell Choi

**Affiliations:** ^1^ Department of Biotechnology College of Life Science and Biotechnology Yonsei University Seoul Republic of Korea; ^2^ CK Regeon Inc. Seoul Republic of Korea; ^3^ Department of Internal Medicine Yonsei University Seoul Republic of Korea; ^4^ Department of surgery Yonsei University College of Medicine Seoul Republic of Korea; ^5^ Korea Mouse Phenotyping Center Seoul National University Seoul Republic of Korea

**Keywords:** adipose tissue remodelling, CXXC5, metabolic diseases, pancreatic β‐cell regeneration, Wnt/β‐catenin pathway

## Abstract

**Background:**

Metabolic diseases, including type 2 diabetes, have long been considered incurable, chronic conditions resulting from a variety of pathological conditions in obese patients. Growing evidence suggests the Wnt/β‐catenin pathway is a major pathway in adipose tissue remodelling, pancreatic β‐cell regeneration and energy expenditure through regulation of key metabolic target genes in various tissues. CXXC5‐type zinc finger protein 5 (CXXC5) is identified negative feedback regulator of the Wnt/β‐catenin pathway that functions via Dishevelled (Dvl) binding.

**Methods:**

Expression level of CXXC5 was characterised in clinical samples and diabetes‐induced mice model. Diabetes‐induced mice model was established by using high‐fat diet (HFD). HFD‐fed mice treated with KY19334, a small molecule inhibiting CXXC5‐Dvl protein–protein interaction (PPI), was used to assess the role of CXXC5 in metabolic diseases.

**Results:**

Here, we show that CXXC5 is overexpressed with suppression of Wnt/β‐catenin signalling in visceral adipose tissues of patients with obesity‐related diabetes. Meanwhile, *Cxxc5^−/−^* mice fed an HFD exhibited resistance to metabolic dysregulation. KY19334 restores the lowered Wnt/β‐catenin signalling and reverses metabolic abnormalities as observed in HFD‐fed *Cxxc5^−/−^* mice. Administration of KY19334 on HFD‐fed mice had a long‐lasting glucose‐controlling effect through remodelling of adipocytes and regeneration of pancreatic β‐cells.

**Conclusion:**

Overall, the inhibition of CXXC5 function by small molecule‐mediated interference of Dvl binding is a potential therapeutic strategy for the treatment of obesity‐related diabetes.

## Graphical abstract

- CXXC5 is abundantly expressed in the adipose tissues from obese‐T2DM patients.
- Ablation of *Cxxc5* resists obesity‐related metabolic diseases.
- KY19334, a small molecule blocking CXXC5‐Dvl binding, reverses obesity‐related metabolic diseases with adult tissue remodelling.
- Effects on glycaemic control with KY19334 prolong during post‐treatment.

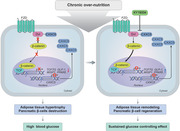

## 1 INTRODUCTION

Type 2 diabetes mellitus (T2DM) is one of the major metabolic diseases. Currently, clinically available drugs for T2DM, including sulfonylureas, glucagon‐like peptide‐1 (GLP‐1) agonists, sodium‐glucose transport protein 2 (SGLT2) inhibitors, peroxisome proliferator‐activated receptor gamma (PPARγ) agonists, dipeptidyl‐peptidase 4 (DPP4) inhibitors and biguanides, control blood glucose levels by acting on peripheral insulin target tissues, such as pancreas, intestine, muscle and liver.^1^, ^2^ Despite recent advances in therapeutics, the glucose‐controlling effect of these drugs is transient; thus, patients need to be prescribed these drugs daily throughout their lifetime.^3^

Growing evidence indicates that the Wnt/β‐catenin signalling is a major player in the regeneration of tissues that have been damaged by pathological conditions.^4^, ^5^, ^6^ The direct Wnt/β‐catenin signalling target genes, such as transcription factor 7 like 2 (*TCF7L2*), wnt1‐induced secreted protein 1 (*WISP1*)*, GLP1* and peroxisome proliferator‐activated receptor delta (*PPARδ*), play major roles in obesity and diabetes.^7^ Especially, TCF7L2 promotes β‐cell regeneration via stimulating proliferation and differentiation, as well as improves insulin secretion in islets.^8^ Also, a novel adipokine WISP1 mediates the anti‐adipogenic activities by suppression of low‐grade inflammatory cytokines in adipose tissues that leads to adipose tissue remodelling.^9^

The role of Wnt/β‐catenin signalling inactivation in the pathogenesis of T2DM was further indicated by the elevation of Dickkopf‐1 (DKK‐1), a Wnt/β‐catenin signalling antagonist, in the serum of a patient with T2DM.^10^, ^11^ Inactivation of Wnt/β‐catenin signalling modifies the adipokine‐secretion profile followed by obesity‐induced adipose tissue inflammation, thereby influencing systemic insulin resistance.^6^, ^12^ Activation of Wnt/β‐catenin pathway by inhibition of glycogen synthase kinase 3 beta (GSK3β) has been known for treatment of T2DM; however, the blood‐glucose‐lowering effects by GSK3β inhibitor are transient because its effects are confined to the liver.^13^ Therefore, identifying a factor regulating the Wnt/β‐catenin pathway, especially which drives metabolic diseases, is important for the systemic improvement in diverse metabolic diseases, including diabetes.

We found that Wnt/β‐catenin signalling is suppressed in adipose tissues of patients with obesity‐related T2DM and adipose and liver tissues of HFD‐induced diabetic mice. Therefore, identifying an endogenous factor suppressing Wnt/β‐catenin signalling and restoration of Wnt/β‐catenin signalling by a specific blockade of its functions could be a therapeutic strategy for metabolic diseases.

CXXC5‐type zinc finger protein 5 (CXXC5) is identified negative feedback regulator of the Wnt/β‐catenin pathway that functions via Dishevelled (Dvl) binding.^14^, ^15^ The functional role of CXXC5 as a driving factor in metabolic diseases was indicated by the induction of Cxxc5 in adipose and liver tissues of HFD‐fed obese mice. The C*xxc5^−/−^* mice resisted obesity and obesity‐related insulin resistance when fed an HFD.

To confirm the potential role of Cxxc5 as a target for the treatment of obesity and diabetes, we orally administered KY19334, a small molecule that specifically interferes with the cytosolic function of CXXC5 as a negative regulator of the Wnt/β‐catenin pathway by inhibition of the CXXC5‐Dvl protein–protein interaction (PPI),^16^ and examined its effects on metabolism. As for the HFD‐fed *Cxxc5^−/−^* mice, HFD‐fed *Cxxc5^+/+^* mice administered KY19334 revealed a reduction in abnormal metabolic phenotypes, such as obesity, insulin resistance and other diabetes‐related phenotypes. Differently with sitagliptin, which is a frequently prescribed drugs for T2DM patients, KY19334 showed sustained effects in controlling fasting glucose levels in HFD‐fed mice. The therapeutic effect of KY19334 on glucose control was not caused by direct blood glucose control but by the regeneration and subsequent remodelling of the damaged tissues caused by the disease state. The therapeutic effects of KY19334 on metabolic diseases phenotypes were acquired by restoration of suppressed Wnt/β‐catenin pathway via blockade of the functions of the aberrantly overexpressed Cxxc5.

Overall, the restoration of the metabolic phenotypes with prolonged control of blood glucose levels by the CXXC5‐Dvl PPI inhibitor provides a potential approach for the treatment of insulin resistance and metabolic diseases.

## 2 MATERIALS AND METHODS

### 2.1 Human visceral fat tissue specimens

To monitor the expression patterns of β‐catenin and CXXC5 during the development of obesity‐related diabetes, 5‐mm biopsy specimens were obtained from the liver or colon of patients with cancer who had undergone surgery. The age of the subjects ranged from 43 to 82 years, and their BMI was between 17 and 32 kg/m^2^. Individuals were divided into four cohorts according to BMI and DM grade (lean, BMI < 25 and DM grade = 0, 1, 2) divided into four cohorts. (i) lean, BMI < 25, DM = 0; (ii) obese, BMI > 25, DM = 0 or 1; (iii) lean, BMI < 25, DM = 2 and (iv) obese, BMI > 25, DM = 2. Experiments using patient samples were approved by the Institutional Review Board of the Clinical Research Institute of Severance Hospital and were conducted according to the Declaration of Helsinki Principles.

### 2.2 Animals

The generation of *Cxxc5^−/−^* mice has been described previously.^17^ *Cxxc5* heterozygous mice were intercrossed for four generations to obtain littermate wild‐type and *Cxxc5^−/−^* mice and were maintained on a C57BL/6 background. Six‐week‐old *Cxxc5^+/+^* and *Cxxc5^−/−^* mice were fed an HFD for 8 weeks.

Wild‐type male C57BL/6 mice (KOATECH, Seoul, Korea) were fed an HFD consisting of 60% calories from fat (Research Diet, D12492) for 18 weeks. To validate the successful establishment of the insulin‐resistant mouse model, fasting glucose levels were assessed using a One Touch Ultra glucometer (LifeScan). Subsequently, each HFD‐fed mouse with fasting glucose levels higher than 7.0 mmol/L was orally administered KY19334 (25 mg/kg), sitagliptin (50 mg/kg, Selleckchem) or its vehicle^18^ for 5 days at weeks 8 and 12. After the removal of the drugs, mice were maintained for 3 weeks on an HFD.

To monitor pancreas regeneration, six‐week‐old *Cxxc5^+/+^* and *Cxxc5^−/−^* mice were fed an HFD as described above. After 4 weeks of dietary treatment, the mice were intraperitoneally injected with STZ (50 mg/kg/day) for 1 week, and the control mice were injected with saline. After 2 weeks, *Cxxc5^+/+^* mice with non‐fasting glucose levels higher than 16.7 mmol/L were orally administered KY19334 (25 mg/kg/day) or sitagliptin (50 mg/kg/day) or its vehicle by oral gavage for 4 weeks.

For the glucose tolerance test (GTT) or insulin tolerance test (ITT), mice were injected with D‐glucose (1.5 g/kg body weight) after 16 h starvation or human insulin (0.75 U/kg body weight) after 4 h starvation, respectively. Tail blood was drawn at 0, 15, 30, 60, 120 and 180 min intervals, and blood glucose levels were measured using a One Touch Ultra glucometer (LifeScan). All mice were maintained under temperature‐controlled and light‐controlled (standard 12 h light/dark cycle) conditions and provided with food and water ad libitum. All protocols were reviewed and approved by the Institutional Review Board of Severance Hospital, Yonsei University College of Medicine (09‐013).

### 2.3 Blood chemistry

Total blood of mice was collected by cardiac puncture after fasting. The blood was allowed to clot for 30 min and then centrifuged at 1000 *× g* for 10 min to obtain the supernatant, with which to measure metabolic parameters in the serum. ELISA assay kits were used to assess serum insulin (Millipore, EZRMI‐13K), glucagon (Millipore, EZGLU‐30L), FFA (Cayman Chemical, 700310), adiponectin (ABclonal, RK02574), active GLP‐1 (7‐36) (EDI, KT871) and c‐peptide (ALPCO, 80‐CPTMS‐E01). The insulin function test was evaluated using HOMA‐IR. Serum chemistry variables, including total cholesterol, HDL‐cholesterol, glucose, TG, ALT and AST concentrations, were measured using a blood chemistry analyser (FUJI DRI‐CHEM 4000i). The calibration of serum parameters was performed using the quality control card supplied with the FUJI DRI‐CHEM slides whenever slides from a new lot were used.

### 2.4 Adipokine‐related protein analyses

Adipokines and hormones in mouse serum were measured using mouse adipokine array kits (Proteome Profiler and Human Cytokine; R&D System, ARY013), allowing simultaneous detection of the expression levels of 38 different obesity‐related proteins. The array was performed according to the manufacturer's instructions. Blots were developed with enhanced chemiluminescence using a luminescent image analyser LAS‐3000 (Fujifilm). All data were normalized by the intensity of the reference spots in each membrane following the manufacturer's instructions.

### 2.5 Haematoxylin and eosin (H&E) staining

The dissected tissues were fixed in 4% neutral paraformaldehyde and embedded in paraffin. Paraffin sections were cut to a thickness of 4 μm. The sections were deparaffinized in three changes of xylene and rehydrated using a graded ethanol series. The sections were stained with haematoxylin for 7 min and stained with eosin for 2 min. The adipocyte cell size was measured in 20 randomly chosen microscopic areas from 3 independent animals using a Nikon bright‐field optical microscope (Nikon TE‐2000U). The average size of the adipocytes was determined using ImageJ software.

### 2.6 Immunohistochemical (IHC) analysis

Paraffin sections were deparaffinized and rehydrated. For antigen retrieval, slides were autoclaved in 10 mM sodium citrate buffer (pH 6.0). Sections were blocked in phosphate‐buffered saline (PBS) containing 10% BSA at 20°C for 30 min. The sections were incubated overnight at 4°C with the following dilutions of primary antibodies: anti‐β‐catenin (1:100, 610514, BD, NJ), anti‐β‐catenin (1:100, ab16051, Abcam, Cambridge, UK), anti‐CXXC5 (1:50, Lab made), anti‐F4/80 (1:100, sc‐377009, Cell Signaling Technology, MA), anti‐CD11b (1:100, ab133357, Abcam), anti‐insulin (1:1000, I2018, Sigma‐Aldrich), anti‐PCNA (1:100, sc‐56, Santa Cruz Biotechnology, Inc., TX) and anti‐pancreatic and duodenal homeobox 1 (Pdx‐1; 1:400, 5679S, Cell Signaling Technology). The slides were washed with PBS, incubated with Alexa Fluor 488‐ (1:300, A11001, Invitrogen, MA) or Alexa Fluor 555‐conjugated IgG secondary antibodies (1:300, A21428, Invitrogen) at 20°C for 1 h, and counterstained with DAPI (1:5000, D9564, Sigma‐Aldrich). The images were captured using an LSM700 META confocal microscope (Carl Zeiss, Jena, Germany) after excitation with 405, 488 or 543 nm laser lines. To block endogenous peroxidase activity before peroxidase IHC analysis, tissues were incubated with 1% H_2_O_2_ (Samchun Chemicals, Gyeonggi, Korea) for 10 min. Before incubating the sections with mouse primary antibody, mouse IgG was blocked using a M.O.M. Mouse IgG blocking kit (Vector Laboratories, CA). Sections were incubated with primary antibodies overnight at 4°C with the following dilution with the primary antibody, anti‐uncoupling protein 1 (UCP1; 1:100, ab10983, Abcam). Sections were then incubated with biotinylated anti‐rabbit (1:300, BA 1000, Dako, Hamburg, Germany) secondary antibodies for 1 h at 20°C. The samples were stained with 3,3‐diaminobenzidine (DAB; Dako) for 3–7 min and counterstained with Mayer's haematoxylin (Muto, Tokyo, Japan). All incubations were conducted in a humid chamber. Signals were analysed using a bright‐field microscope (Nikon TE‐2000U).

### 2.7 Metabolic monitoring and body composition analysis

Metabolic performance (energy intake and energy expenditure) was studied using a PHENOMASTER automated combined indirect calorimetry system (TSE system GmBH). The mice were acclimated for 24 h in a metabolic chamber and were provided with food and water. They were evaluated for 3 days to measure oxygen consumption (VO_2_), carbon dioxide production (VCO_2_), ambulatory counts and the respiratory exchange ratio. Additionally, an LF50 body composition analyser (Bruker) was used to determine the body composition (lean body mass and total body fat) of the mice. The temperature for these studies was maintained at 22°C with a 12 h light/dark cycle. Standard in‐house software was used for energy expenditure.

### 2.8 Bioinformatic data analyses

Molecular pathway dysregulation in the human visceral adipose tissues was determined by gene set enrichment analysis, surveying the molecular pathway gene set in the Molecular Signature Database (MsigDB) (www.broadinstitute.org/msigdb). Cross‐species comparison of transcriptomic dysregulation was performed in the space of the molecular pathway gene sets from HALLMARK and KEGG databases, and statistically significant dysregulation was defined as a false discovery rate (FDR) < 0.01 in either of the two human visceral and subcutaneous adipose tissue transcriptome datasets: normal (*n* = 5) vs. T2DM (*n* = 5) subjects (GSE16415), normal glucose tolerance (*n* = 17) vs. T2DM (*n* = 17) subjects (hgu‐133a), lean (*n* = 10) vs. obese (*n* = 10) subjects (GSE2508), normal glucose tolerance (NGT) (*n* = 4) vs. impaired glucose tolerance (IGT) (*n* = 4) vs. T2DM (*n* = 4) subjects (GSE27951) and insulin‐sensitive (*n* = 5) vs. resistant (*n* = 5) subjects (GSE15773).

### 2.9 Triglyceride (TG) assay

Tissue samples were homogenised in the Standard Diluent Assay Reagent provided by a Triglyceride Colorimetric Assay kit (Cayman Chemical, Ann Arbor, MI) and triglyceride levels were quantified based on reference standards as described in the manufacturer's instructions.^19^

### 2.10 Quantitative real‐time polymerase chain reaction (PCR)

Total RNA was extracted from ground tissue powder using the TRIzol reagent (Invitrogen), according to the manufacturer's instructions. Reverse transcription was performed with M‐MLV reverse transcriptase (Invitrogen) using 2 μg of total RNA. Synthesised cDNA was diluted to a concentration of 1 μg. Quantitative PCR analyses were performed in a Rotor‐Gene Q real‐time PCR cycler (Qiagen) using SYBR green reagent (Qiagen) as follows: 95°C for 10 min followed by 40 cycles at 95°C for 5 s, and 60°C for 15 s. Relative quantification of mRNA levels was performed using the comparative Ct method (∆∆Ct). All mRNA values were normalised to those of *GAPDH*. The primer sequences are listed in Table S1.

### 2.11 Western blotting

Cells and tissues were lysed using radioimmunoprecipitation assay (RIPA) buffer (150 mM NaCl, 10 mM Tris, pH 7.2, 0.1% SDS, 1.0% Triton X‐100, 1% sodium deoxycholate and 5 mM EDTA). Samples were separated on 12% SDS polyacrylamide gels and transferred onto PROTRAN nitrocellulose membranes (Shleicher and Schuell, Co., NH). After blocking with PBS containing 5% non‐fat dry skim milk and 0.07% Tween 20, the membranes were incubated with antibodies specific for β‐catenin (1:1000, sc‐7963, Santa Cruz Biotechnology, Inc.), CXXC5 (1:500, Lab made) and β‐actin (1:1000, ab8226, Abcam) at 4°C for 12 h. Membranes were then incubated with horseradish peroxidase‐conjugated anti‐rabbit (Bio‐Rad, CA) or anti‐mouse (Cell Signaling Technology) IgG secondary antibody. Protein bands were visualized with enhanced chemiluminescence (GE Healthcare, IL) using a luminescent image analyser (LAS‐3000).

### 2.12 Oil Red O staining

Liver tissues were washed with PBS and 70% isopropanol (Duksan Pure Chemicals) and stained with Oil Red O solution (Sigma‐Aldrich) at 20°C for 12 h. The samples were thoroughly washed with distilled water. The tissues were counterstained with Mayer's haematoxylin. Images of Oil Red O staining were visualized using a bright‐field microscope (Nikon TE‐2000U). For the quantification of lipid content, Oil Red O was eluted by adding 500 μl isopropanol containing 4% nonidet P‐40 to each well, and the absorbance was measured spectrophotometrically at 590 nm.

### 2.13 Isolation of primary pancreatic islet cells

Primary islet cells from C57BL/6 mice fed NCD or HFD with STZ injection were isolated and cultivated as described.^20^ Briefly, the pancreas was digested in Hanks' balanced salt solution (HBSS; Gibco) containing 1 mg/ml collagenase‐P (Roche) for 15 min at 37°C. The digestion was stopped by adding HBSS containing 1 mM CaCl_2_. The pellets were resuspended in a pre‐wetted 70 μm cell strainer and the captured islets were maintained in RPMI 1640 (Gibco) containing 10% FCS, 20 mM L‐glutamine (Gibco) and 100 U/L penicillin/streptomycin (Gibco) at 37°C in a 5% CO_2_ environment.

### 2.14 Transfection, drug treatment and fluorescence staining of primary islet cells

Isolated islets were seeded in 24‐well plates and maintained in RPMI containing 10% FCS, 20 mM L‐glutamine, and 100 U/L penicillin/streptomycin with or without KY19334. For transient transfection, islets were transfected with siRNA using lipofectamine (Invitrogen) in Opti‐MEM (Gibco). Islet cells were fixed with 4% paraformaldehyde in PBS for 30 min at 20°C. The cells were then permeabilized and blocked with 0.5% Triton X‐100 and 5% BSA in PBS for 30 min at 20°C. Cells were then incubated overnight at 4°C with the following dilutions of primary antibodies: anti‐insulin (1:500, I2018, Sigma‐Aldrich) and anti‐Ki67 (1:100, ab15580, Abcam). Additionally, the cells were incubated with Alexa Fluor 488‐conjugated (1:300, A‐11001, Invitrogen) or Alexa Fluor 555‐conjugated IgG secondary antibody (1:300, A‐21428, Invitrogen) at 20°C for 1 h and counterstained with DAPI (1:5,000, D9564, Sigma‐Aldrich). The stained cells were captured using an LSM700 META confocal microscope (Carl Zeiss) after excitation with 405, 488 or 543 nm laser lines.

### 2.15 Glucose‐stimulated insulin secretion (GSIS) assay

After 3 days of transfection or drug treatment, islets were incubated in KRBH buffer containing 10 mM HEPES, 20 mM NaHCO_3_, 0.2% BSA and 2.8 mM glucose for 1 h for equilibration. To measure GSIS, the equilibration medium was replaced with KRBH buffer enriched with different glucose concentrations (low glucose, 2.8 mM; high glucose, 16.7 mM) and incubated for 1 h at 37°C. At the end of each incubation, the media were collected. Secreted insulin was detected using an insulin enzyme‐linked immunosorbent assay kit (Millipore).

### 2.16 β‐Cell mass measurement

β‐Cell mass from the insulin antibody‐stained sections was measured using HistoQuest software (TissueGnostics). The cross‐sectional area occupied by all β‐cells in the pancreas was quantified. Total β‐cell area and total pancreatic mass for each animal were calculated as the sum of the determinations from each of the 8–10 segments of the pancreas. A total of 1000–1500 β‐cells were counted for each pancreas. Total β‐cell mass for each pancreas was determined as the product of the total cross‐sectional β‐cell area over the total tissue area and the weight of the pancreas before fixation.

### 2.17 Quantitation of signal intensity

For immunofluorescent staining, the intensity was analysed with NIS Elements V3.2 software (Nikon). The blue channel was used as a reference to visualise the nuclei, and the threshold was defined for red, green or blue channels. Mean intensity was calculated in the red and green channels separately, and mean values were estimated from analyses of three independent experiments.

### 2.18 Statistical analysis

Data are presented as the means ± standard deviation (SD). Statistical analyses were performed using an unpaired two‐tailed Student's *t*‐test. Asterisks denote statistically significant differences (*p < 0.05, **p < 0.01, ***p < 0.001).

## 3 RESULTS

### 3.1 CXXC5 is upregulated with suppression of the Wnt/β‐catenin target genes involved in the metabolism of visceral fat tissues in patients with obesity‐related diabetes

**FIGURE 1:**
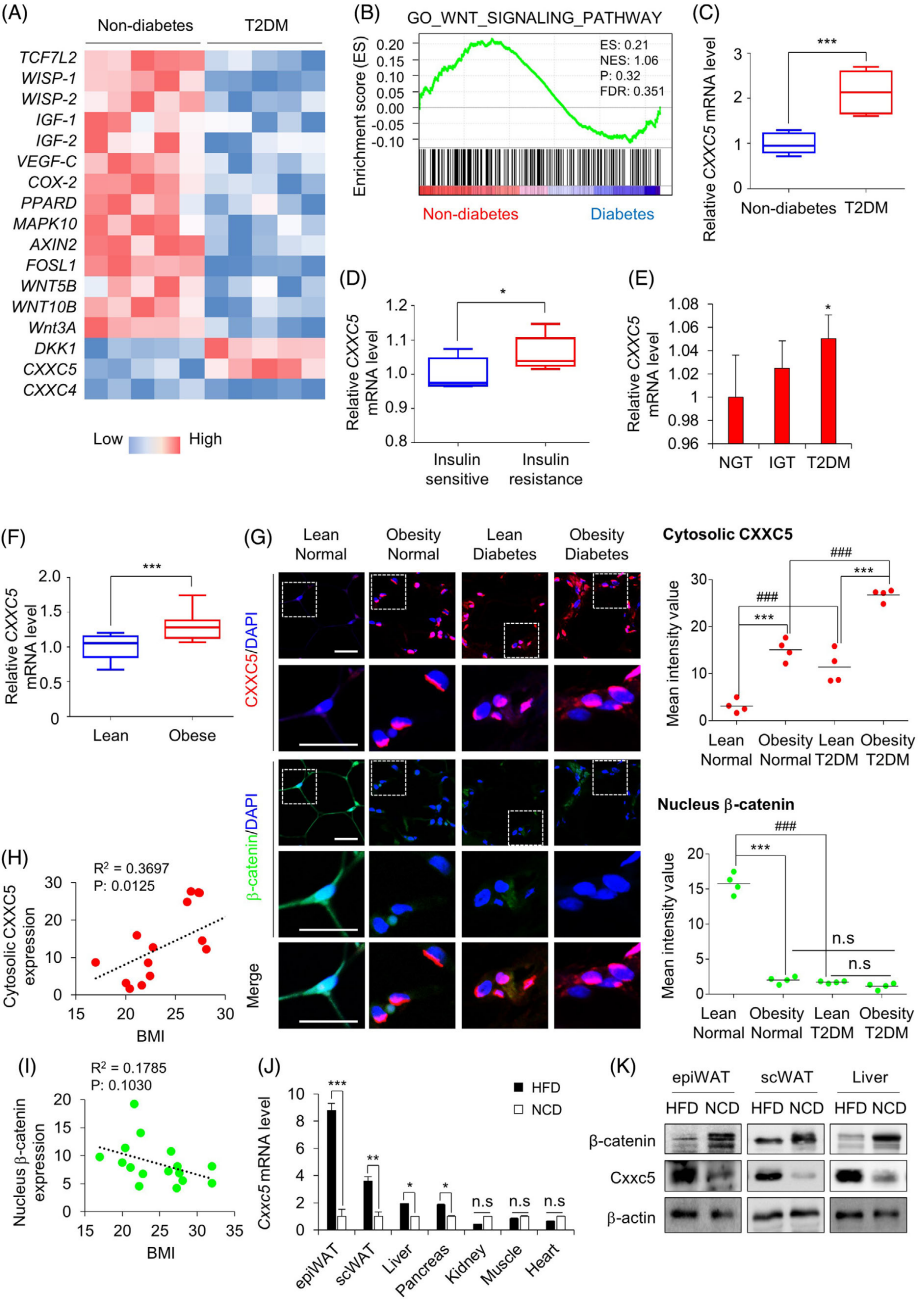
Expression of CXXC5, an inhibitor of the Wnt/β‐catenin pathway, in human adipose tissues of patients with obesity‐induced diabetes. (A) Hierarchical clustering and heat‐map of RNA‐seq data in visceral adipose tissues from women with obesity‐induced diabetes (*n* = 5 for all groups). The colour scale shows Z‐score fragments per kilobase of transcript per million mapped reads representing the mRNA levels of each gene in the blue (low expression) to red (high expression) coloured scheme. (B) Gene set enrichment analysis of microarray transcriptome data from BMI‐matched patients with diabetes for Wnt/β‐catenin signalling‐activated gene signature. Black columns indicate 83 enriched genes in visceral adipose tissues of subjects with normal glucose tolerance or diabetes, involving the Wnt/β‐catenin signalling pathway (*n* = 17 for all groups). NES, normalized enrichment score; ES, enrichment score; FDR, false discovery rate. (C–F) Expression levels of *CXXC5* in visceral adipose tissues from lean and obese subjects (GEO: GSE16415, GSE15773, GSE27951, GSE2508) (C), in visceral adipose tissues from BMI‐matched obese patients suffering from insulin sensitivity and resistance (*n* = 5 per group) (D), in subcutaneous adipose tissues from subjects with normal glucose tolerance (NGT), impaired glucose tolerance (IGT) and T2DM (*n* = 4 per group) (E), in subcutaneous adipose tissues from lean and obese subjects (*n* = 10 per group) (F). Expression levels of mRNA were normalized by non‐diabetes (C), insulin sensitive (D), NGT (E), lean group (F). (G–I) Visceral adipose tissues from human subjects that were lean, obese, diabetic and obese‐diabetic (*n* = 4 per group). Representative IHC images of CXXC5 and β‐catenin in visceral adipose tissue and quantitative analyses of IHC staining for CXXC5 and β‐catenin (G: upper panel). The quantitative mean intensity value of IHC staining of CXXC5 and β‐catenin was performed (G: lower panel), correlation of CXXC5 expression (H) and β‐catenin expression (I) with BMI. (J, K) *Cxxc5^+/+^* mice were fed HFD or NCD for 8 weeks (*n* = 6 per group). Relative *Cxxc5* mRNA expression in epiWAT, scWAT, liver, pancreas, kidney, muscle and heart tissues was normalized by NCD‐fed mice group (J) and β‐catenin and Cxxc5 expression in epiWAT, scWAT and liver tissues (*n* = 3 per group) (K). Scale bars = 100 μm. All data are presented as the mean ± SD. *p < 0.05, ***p < 0.001 determined by Student's *t*‐test. ^#^ *p* < 0.05, ^##^ *p* < 0.01, ^###^ *p* < 0.001 determined by Tukey's post hoc test

**FIGURE 2:**
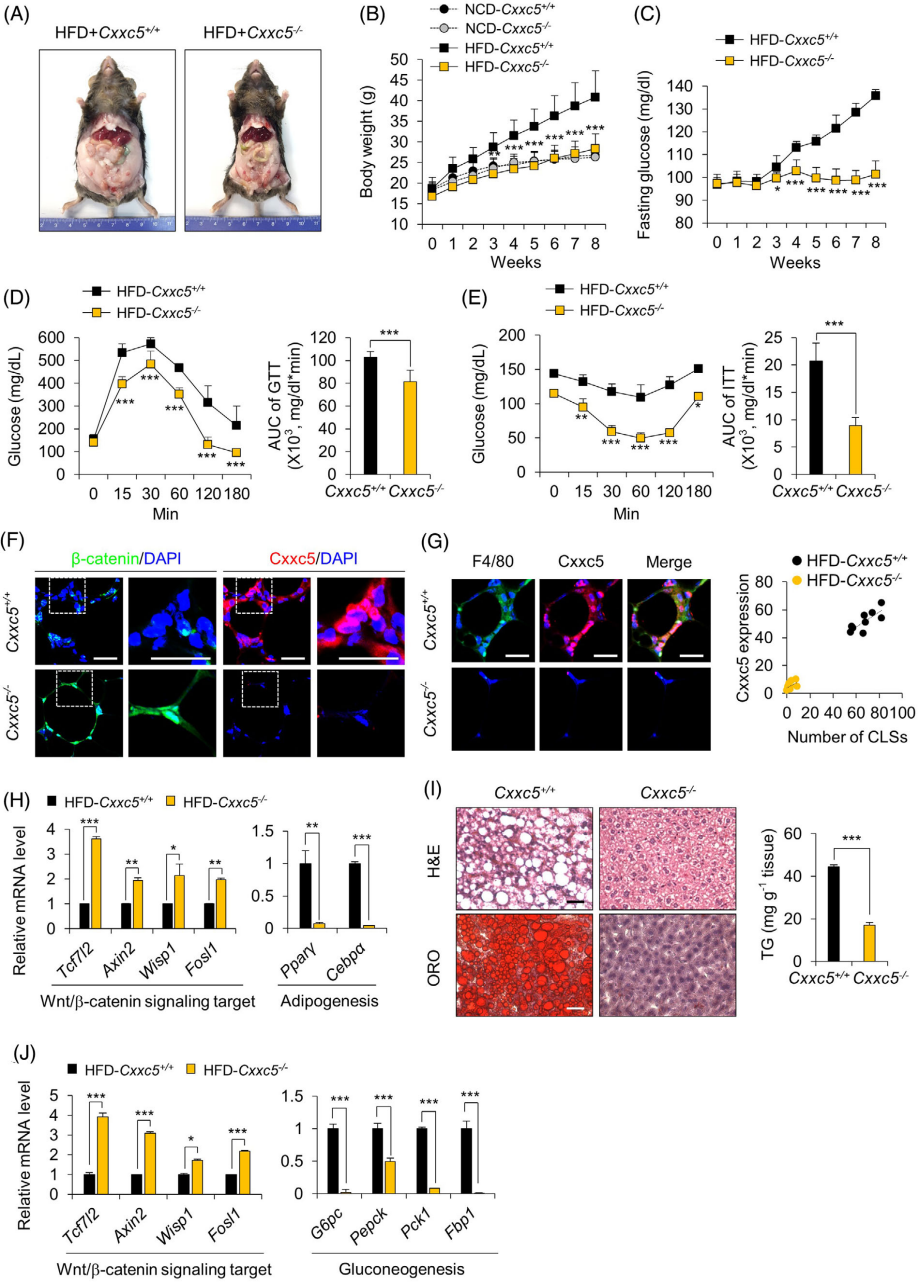
Ablation of *Cxxc5* resists obesity‐related insulin resistance and hepatosteatosis in HFD‐fed mice. (A–E) *Cxxc5^+/+^* and *Cxxc5^−/−^* mice were fed HFD or NCD for 8 weeks (*n* = 9–13 per group). Representative photographs of HFD‐fed *Cxxc5^+/+^* and *Cxxc5^−/−^* mice (A), body weight (B), fasting glucose levels (C), glucose tolerance test (D) and insulin tolerance test (E) and area under the curve (AUC). (F, G) epiWAT from *Cxxc5^+/+^* and *Cxxc5^−/−^* mice fed HFD for 8 weeks (*n* = 9–13 per group). Representative images of IHC staining for β‐catenin, Cxxc5 (F), F4/80 and Cxxc5 (G: left panel) and the correlation of cytosolic Cxxc5 with the number of CLSs (G: right panel). (H) Relative expression levels of Wnt/β‐catenin pathway target (left panel) and adipogenesis (right) genes. Expression levels of mRNA were normalized by HFD‐fed *Cxxc5^+/+^* mice group. (I, J) Liver tissue from *Cxxc5^+/+^* and *Cxxc5^−/−^* mice fed HFD for 8 weeks (*n* = 9–13 per group). Representative images of H&E staining and Oil Red O staining (I: left panel) and TG concentration in the liver tissue (I: right panel) and relative mRNA expression levels of Wnt/β‐catenin signalling target and gluconeogenic genes (J). Expression levels of mRNA were normalized by HFD‐fed *Cxxc5^+/+^* mice group. Scale bars = 100 μm. All data are presented as the mean ± SD. *p < 0.05, **p < 0.01, ***p < 0.001 determined by Student's *t‐*test

The clinical implications of the functional roles of CXXC5 in metabolic diseases and their relationship with Wnt/β‐catenin signalling were investigated in human adipose tissues. As shown by microarray analyses, mRNA levels of the Wnt/β‐catenin signalling target genes related to metabolic diseases, including *TCF7L2*, *WISP‐1*, *IGF‐1* and *PPARδ*, were lower in the visceral adipose tissues from patients with T2DM than in tissues from non‐diabetic subjects (Figure 1A and B). In contrast, the mRNA levels of CXXC5 and DKK‐1, which are negative regulators of the Wnt/β‐catenin signalling, were high in the visceral adipose tissues of patients with obesity‐related T2DM (Figure 1A and C). The increment of CXXC5 mRNA levels in adipose tissues of patients with insulin‐resistant obesity‐related T2DM was confirmed (Figure 1D–F). CXXC5 was highly expressed in subcutaneous and visceral adipose tissues of the obese‐diabetes patients especially at the F4/80+ crown‐like structures (CLSs) of adipose tissues, compared to that in lean non‐diabetic subjects (Figures 1G and S1A and B). However, β‐catenin was expressed in both the nucleus and cytosol of adipose tissue cells of lean non‐diabetic subjects but was mostly abolished in cells from patients with obesity‐related diabetes (Figure 1G). Moreover, CXXC5 and β‐catenin levels were correlated (Figure 1H) and inversely correlated (Figure 1I), respectively, with body mass index (BMI) in visceral fat tissues. The mRNA expression levels of *Cxxc5* were significantly and selectively upregulated, whereas those of Wnt/β‐catenin signalling target genes were downregulated in metabolic target tissues, such as epididymal white adipose tissue (epiWAT), subcutaneous white adipose tissue (scWAT) and the liver of HFD‐fed mice compared to that of NCD‐fed mice (Figures 2J and S2). Additionally, protein levels of Cxxc5 were increased with a decrement of β‐catenin in epiWAT, scWAT and liver tissues in HFD‐fed mice (Figure 2K). Overall, CXXC5 was specifically induced in the subcutaneous and visceral adipose tissues of patients with T2DM.

### 3.2 *Cxxc5^−/−^* mice resist to the development of HFD‐induced obesity and metabolic disease phenotypes

To evaluate the systemic roles of CXXC5 in obesity‐related metabolic diseases, *Cxxc5^+/+^* and *Cxxc5^−/−^* mice were fed either an HFD or NCD for 8 weeks. HFD‐induced obesity did not occur in *Cxxc5^−/−^* mice (Figures 2A and S3A), and their body weights were similar to those of *Cxxc5^+/+^* mice fed NCD (Figure 2B) without alterations in food intake (Figure S3B). Consistent with reduced obesity, HFD‐fed *Cxxc5^−/−^* mice exhibited markedly improved glucose tolerance and insulin sensitivity (Figure 2C–E), with improved levels of metabolic parameters, including leptin, resistin, adiponectin, TGs, total cholesterol and HDL‐cholesterol levels (Figure S4). In contrast, on the NCD, neither *Cxxc5^+/+^* nor *Cxxc5^−/−^* mice exhibited differences in insulin sensitivity, serum levels of glucose, TGs and adipose tissue weight (Figure S5A–D). The roles of the Wnt/β‐catenin signalling suppressor Cxxc5 in metabolic disease‐related phenotypes were confirmed in the epiWAT of HFD‐fed *Cxxc5^−/−^* mice, which had smaller adipocytes and decreased expression levels of F4/80, Cd11b proinflammatory markers in the CLSs with decreased and increased expression of M1 and M2 macrophage markers, respectively (Figures 2F and G and S6A and B). On the HFD, in epiWAT, expression levels of the Wnt/β‐catenin signalling target genes were higher, whereas those of the late adipocyte differentiation markers, such as *Pparγ* and *Cebpα* were decreased in *Cxxc5^−/−^* mice compared to those in *Cxxc5^+/+^* mice (Figure 2H). Differently with *Cxxc5^+/+^* mice, *Cxxc5^−/−^* mice did not exhibit significant hepatic steatosis induced by the HFD (Figure 2I). The expression levels of gluconeogenic genes were markedly reduced with the increment of Wnt/β‐catenin signalling target genes in the livers of HFD‐fed *Cxxc5^−/−^* mice (Figure 2J). Thus, HFD‐induced adipocyte hypertrophy and hepatic steatosis were abrogated in *Cxxc5^−/−^* mice.

### 3.3 KY19334 resulted in sustained glucose control with inhibition of the HFD‐induced metabolic disease phenotypes

To examine whether the blockade of CXXC5 function, especially its interaction with Dvl in the cytosol, could restore Wnt/β‐catenin signalling and exert anti‐diabetic properties, we selected 5‐methoxyindirubin‐3′‐oxime (KY19334), one of the small molecules that activates Wnt/β‐catenin signalling by releasing the CXXC5‐mediated negative feedback regulation. KY19334 is a derivative of indirubin‐3‐oxim (I3O), which was screened using an in vitro high‐throughput screening system to monitor CXXC5‐Dvl PPI and secondarily TOPFlash Wnt reporter activity, followed by chemical synthesis of 80 analogues for improvement.^16^

**FIGURE 3:**
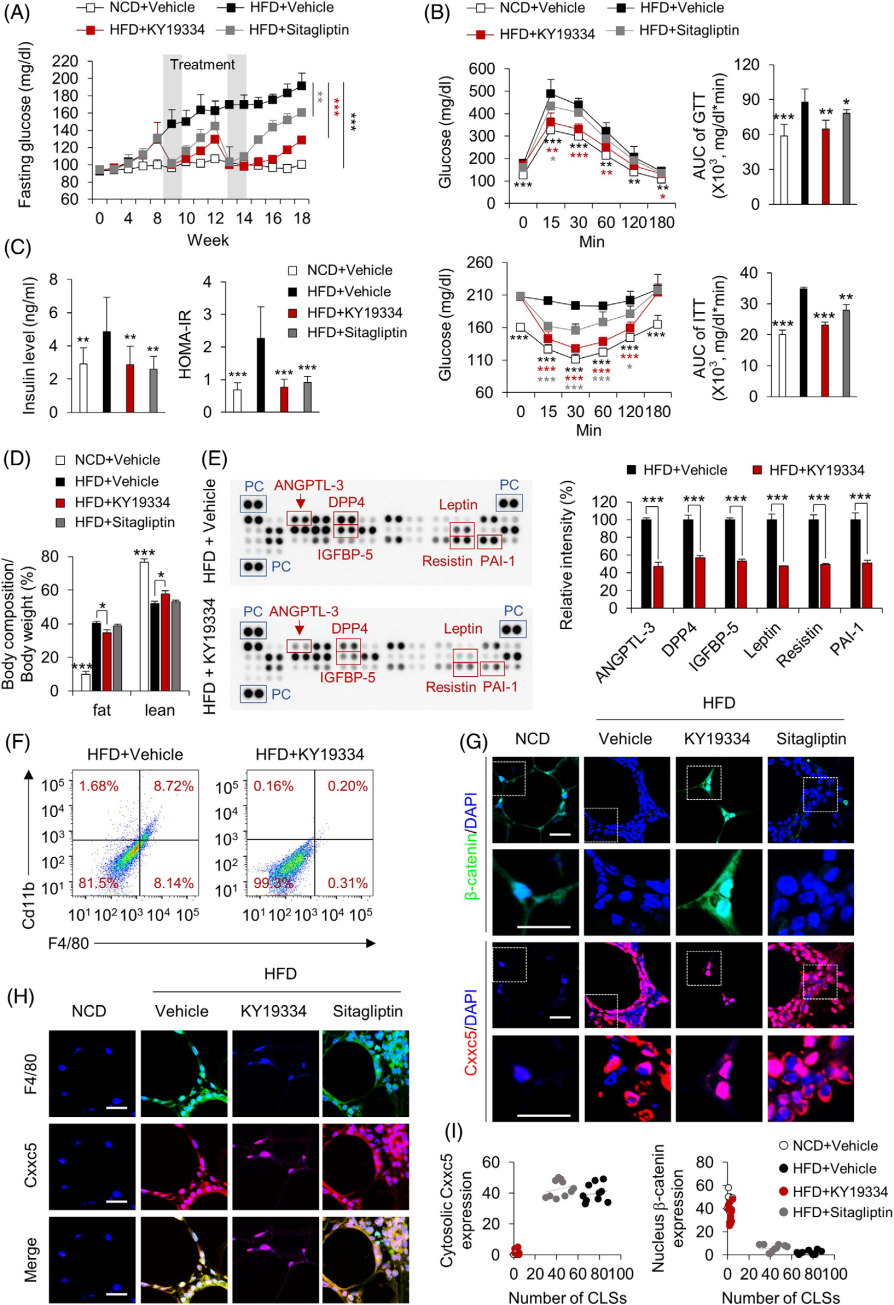
Treatment with KY19334, CXXC5‐Dvl PPI inhibitor, improves obesity‐related insulin resistance with long‐lasting effect. C57BL/6 mice fed NCD or HFD for 18 weeks were p.o. administered KY19334 (25 mg/kg/d) or sitagliptin (50 mg/kg/d) for 5 days on weeks 8 and 12 (*n* = 10 per group). (A) Fasting glucose. (B) Glucose and insulin tolerance tests and area under the curve (AUC). (C) Plasma insulin concentration in the overnight fasted state (left panel) and HOMA‐IR (right panel). (D) Percentage of fat and lean mass. (E) The mouse adipokine and adipokine‐related protein array in plasma (left panel). The quantification mean intensity values of proteins of angiopoietin‐like protein‐3 (ANGPTL‐3), DPP4, IGFBP‐5, Leptin, Resistin and PAI‐1 (right panel). (F) Flow cytometry analysis of the expression of F4/80 and Cd11b and percentage of F4/80^+^Cd11b^+^ cells are shown. (G) Representative IHC images for β‐catenin, Cxxc5. (H) Representative IHC images for F4/80 and Cxxc5. (I) The correlation of cytosolic Cxxc5 and nucleus β‐catenin expression with the number of CLSs. Scale bars = 100 μm. All data are presented as the mean ± SD. *p < 0.05, **p < 0.01, ***p < 0.001 determined by Student's *t*‐test

Oral administration of KY19334 (25 mg/kg) suppressed fasting glucose levels of HFD‐fed mice as effectively as the representative drug sitagliptin at 50 mg/kg, the optimal dosages.^21^ The fasting glucose level was effectively decreased by KY19334 treatment, as it was by sitagliptin, but re‐increment of glucose level after the termination of KY19334 administration was much lower than that shown by treatment of sitagliptin (Figure 3A). The glucose‐controlling effect of KY19334, but not sitagliptin, persisted for 3 more weeks after the second application (Figure 3A). This prolonged effect was not observed in the sitagliptin treatment groups (Figure 3A). KY19334 treatment significantly improved systemic glucose tolerance and insulin resistance compared with sitagliptin treatment in HFD‐fed mice (Figure 3B and C). Consistent with the improvement in insulin resistance by KY19334 treatment in HFD‐fed mice, body weight was decreased without significant changes in food intake (Figure S7A and B). The inhibition of HFD‐induced body weight gain by KY19334 treatment was attributed to reducing overall fat mass, especially in adipose and liver tissues (Figures 3D and S7C). Additionally, KY19334 showed much better effects than sitagliptin in the improvement of endocrine and metabolic parameters, including total cholesterol, HDL‐cholesterol, TGs and adiponectin levels (Figure S7D and E). As confirmed by protein chip analyses, KY19334 decreased the expression levels of various adipokines such as angiopoietin‐3, DPP4, IGFBP‐5, leptin, resistin and PAI‐1, which are implicated in obesity‐related insulin resistance (Figure 3E). The improved insulin resistance of HFD‐fed mice could have resulted from a reduction in inflammatory responses accompanying tissue remodelling in the epiWAT.^22^ KY19334 treatment significantly decreased the expression levels of F4/80 and Cd11b in CLSs, and the size of adipocytes in epiWAT compared to vehicle‐ and sitagliptin‐treated mice (Figures 3F and S8A and B). The cytosolic Cxxc5 was highly expressed with decreased expression of β‐catenin in the F4/80+ CLSs (Figure 3G and H), whereas nuclear β‐catenin was elevated after KY19334 treatment (Figure 3G and I). The mRNA expression levels of all M1 and M2 macrophage marker genes were decreased and increased, respectively, in the epiWAT of KY19334‐treated mice (Figure S8C). Additionally, the Wnt/β‐catenin signalling target genes were upregulated with the downregulation of lipogenesis genes in the epiWAT of KY19334‐treated mice (Figure S8D and E).

### 3.4 KY19334 reduces hepatic steatosis and improves glucose homeostasis

**FIGURE 4:**
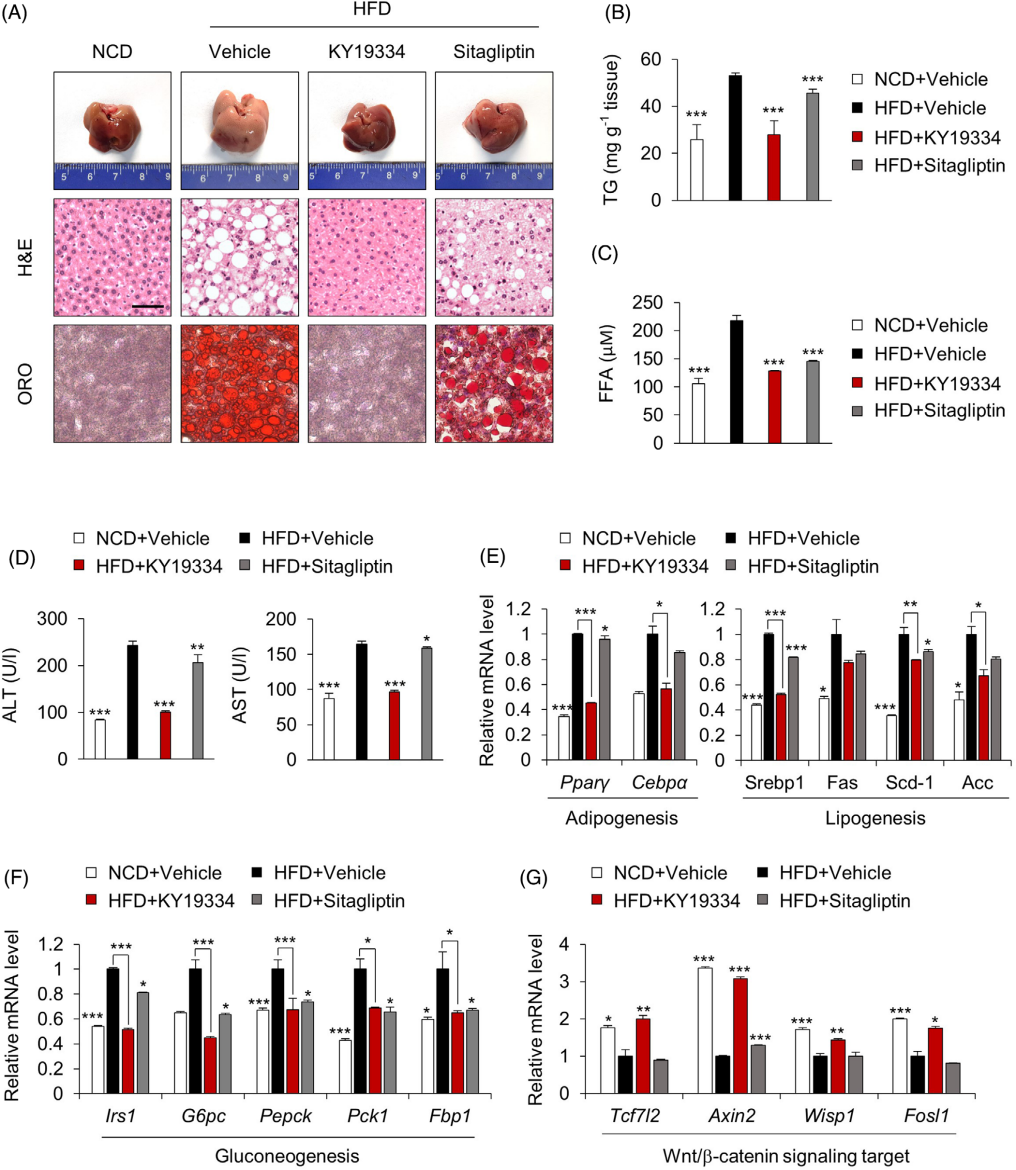
KY19334 treatment improves hepatic glucose homeostasis. C57BL/6 mice fed NCD or HFD for 18 weeks were p.o. administered KY19334 (25 mg/kg/d) or sitagliptin (50 mg/kg/d) for 5 days on weeks 8 and 12 (*n* = 10 per group). (A) Representative images (*n* = 10 independent experiments) of the liver, H&E staining and Oil Red O staining. (B) TG concentration in the liver tissues. (C) Plasma concentration of FFA. (D) Plasma concentration of ALT and AST. (E–G) Relative mRNA expression of lipogenesis (E), gluconeogenesis (F) and Wnt/β‐catenin signalling target genes (G) was normalized by vehicle‐treated HFD mice group. All data are presented as the mean ± SD. *p < 0.05, **p < 0.01, ***p < 0.001 determined by Student's *t*‐test

Hypertrophic adipose tissue releases excess FFAs, taken up by hepatocytes and stored as TGs, resulting in hepatic steatosis and insulin resistance.^23^ Liver tissues from KY19334‐treated mice on the HFD did not form lipid droplets and had reduced hepatic TG levels (Figure 4A). The HFD‐induced increase in factors indicating liver damage, such as ALT, AST and FFAs, was mostly suppressed by KY19334 treatment, and these liver protective effects were more significant than those shown by sitagliptin treatment (Figure 4B–D). Consistent with the histological data, expression levels of lipogenesis and gluconeogenesis genes were suppressed with the induction of Wnt/β‐catenin signalling target genes in the livers of KY19334‐treated mice (Figure 4E–G). Overall, KY19334 improved systemic metabolic homeostasis accompanied by restoration of tissues similar to the intact form and suppressed inflammation, which were not achieved by sitagliptin.

### 3.5 KY19334 enhances energy expenditure by increasing the thermogenic activity of beige‐fat tissues

**FIGURE 5:**
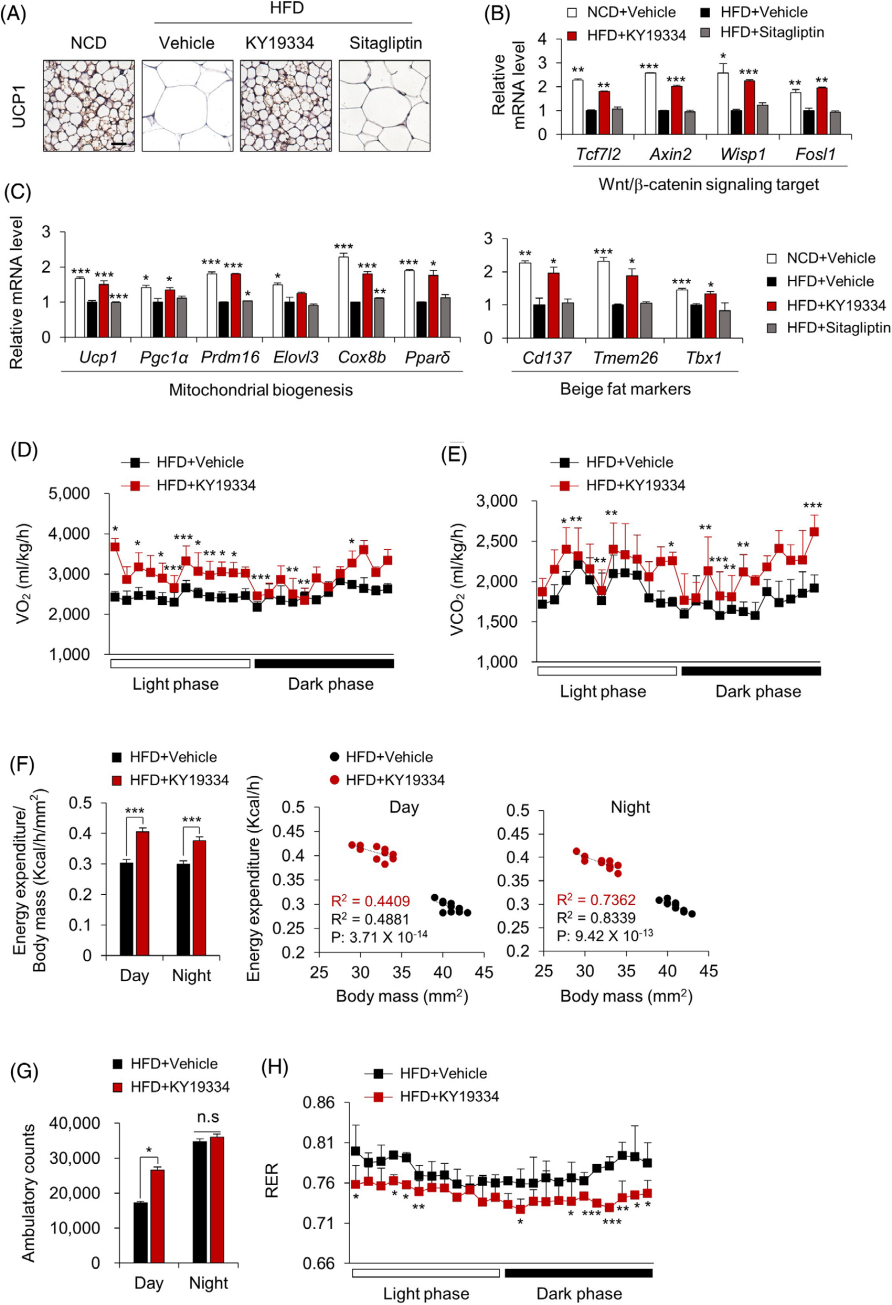
KY19334 treatment increases energy expenditure. C57BL/6 mice fed NCD or HFD for 18 weeks were p.o. administered KY19334 (25 mg/kg/d) or sitagliptin (50 mg/kg/d) for 5 days on weeks 8 and 12 (*n* = 10 per group). (A) Representative images (three total images per group) of UCP1 immunohistochemistry in scWAT. (B, C) Relative mRNA expression levels of Wnt/β‐catenin signalling target genes (B), mitochondrial biogenesis and beige fat markers (C) were normalized by vehicle‐treated HFD mice group. (D) Oxygen consumption. (E) Carbon dioxide production. (F) Energy expenditure normalized for body mass (left panel). Correlation of energy expenditure by body mass using covariance (middle and right panel). (G) Cumulative ambulatory counts. (H) Respiratory exchange ratios. Scale bars = 100 μm. All data are presented as the mean ± SD. *p < 0.05, **p < 0.01, ***p < 0.001 determined by Student's *t*‐test

The KY19334 effects in tissue remodelling were further shown by the browning of adipocyte tissue with an increment of the mitochondrial biogenesis markers, including the major thermogenin UCP1 and beige‐fat markers, and the Wnt/β‐catenin signalling target genes involved in energy metabolism in the scWAT of mice fed the HFD (Figure 5A–C). The stimulation of adipose tissue browning without alteration in food intake indicated a potential enhancement in energy consumption in KY19334‐treated mice on an HFD. KY19334‐treated mice consumed higher levels of VO_2_ and exhaled higher levels of VCO_2_ during both the light and dark periods than those of the vehicle‐treated mice (Figure 5D and E). KY19334‐treated mice also showed higher energy expenditures and more active behavior than vehicle‐treated mice (Figure 5F and G). The lower respiratory exchange ratio of KY19334‐treated mice increased fat usage (Figure 5H). Therefore, blockade of Cxxc5 function promoted energy expenditure by enhancing the thermogenic activities of both brown and beige fats.

### 3.6 KY19334 reverses diabetes phenotypes and promotes pancreatic β‐cell regeneration in HFD and streptozotocin‐treated diabetic mice

**FIGURE 6:**
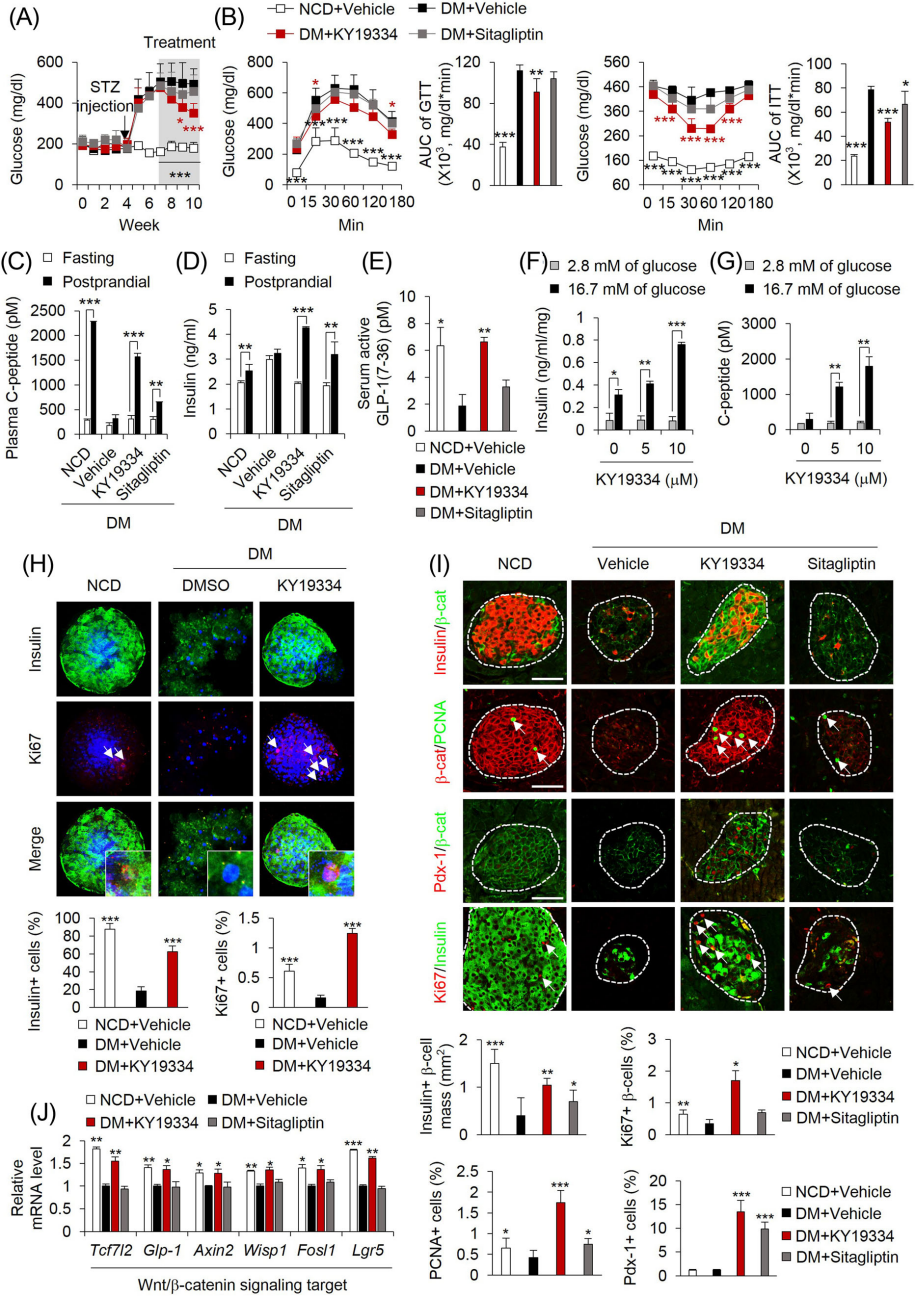
KY19334 treatment restores β‐cell mass and functions in HFD‐fed and STZ‐induced diabetes mellitus (DM) mice. C57BL/6 mice fed NCD or HFD for 4 weeks followed by injection with STZ (50 mg/kg/d) for 1 week. Afterward, mice were p.o. administered KY19334 (25 mg/kg/d) or sitagliptin (50 mg/kg/d) for 4 weeks (*n* = 6 per group). (A) Non‐fasting blood glucose levels. (B) Glucose tolerance and insulin tolerance test and AUC. (C–E) The concentration of plasma C‐peptide (C), insulin (D) and serum active GLP‐1 (E). (F–H) Isolated islets from the pancreas of NCD or DM mice treated with DMSO or KY19334 for 72 h. The concentration of secreted insulin (F) and C‐peptide (G) from islets in response to different concentrations measured after incubation for 1 h with either low (2.8 mM) or high (16.7 mM) glucose in KRBH buffer. (H) Representative images of immunofluorescent staining for insulin and Ki67 (upper panel). Quantitative analyses of insulin‐ and Ki67‐positive cells, respectively, in the islets (lower panel). (I) Representative images of IHC staining of the pancreas for insulin, β‐catenin, PCNA, Pdx‐1 and Ki67. Arrows indicate proliferating β‐cells (upper panel). Quantitative analyses of insulin‐positive β‐cell mass, PCNA, Pdx‐1 and Ki67 positive cells in pancreatic tissues (lower panel). (J) Relative mRNA expression of Wnt/β‐catenin signalling target genes. Expression levels of mRNA were normalized by vehicle‐treated HFD mice group. Scale bars = 100 μm. All data are presented as the mean ± SD. *p < 0.05, **p < 0.01, ***p < 0.001 determined by Student's *t*‐test. DM: Diabetes mellitus; β‐cat: β‐catenin

Considering that loss of functional β‐cells is crucial during late‐stage diabetes,^24^ we investigated whether KY19334 could reverse diabetes by promoting pancreatic β‐cell regeneration in HFD‐fed and the STZ‐induced diabetes mellitus (DM) mouse model.^25^ The STZ‐induced diabetic phenotypes, including hyperglycaemia, insulin resistance, and pancreatic dysfunction, were improved by KY19334, and the effects were more significant than those of sitagliptin (Figure 6A–D). These results of the KY19334 treatment were correlated with enhanced insulin secretion levels (Figure 6D) and plasma levels of active GLP‐1 (Figure 6E), one of the Wnt/β‐catenin pathway target genes, which stimulate insulin secretion from pancreatic β‐cells in a glucose‐dependent manner.^21^ Pancreatic β ‐cells, which were destroyed in diabetic mice, were significantly restored by KY19334, and their functionality was confirmed by glucose‐stimulated insulin and C‐peptide secretion (Figure 6F–H). The expression levels of proliferation markers, Pdx‐1 and insulin were also increased by KY19334 treatment, indicating that the functionality of the regenerated pancreatic β‐cells was enhanced by KY19334 (Figure 6I). The role of KY19334‐induced recovery of Wnt/β‐catenin signalling in the regeneration of pancreatic β‐cells was indicated by the specific increase in β‐catenin levels and Wnt/β‐catenin signalling target genes in the cells (Figure 6I and J). The role of Cxxc5 in the regeneration of functional β‐cells was confirmed by the KY19334 mimetic effects on Cxxc5^−/−^ diabetic mice (Figure S9A–J).

## 4 DISCUSSION

Diabetes is one of the major metabolic diseases related to obesity, and current drugs result in the transient glucose‐controlling effect due to their function through enhancement of glucose uptake into insulin sensitive tissues such as liver and pancreas.^3^

In this study, we suggested a new therapeutic approach that results in a long‐lasting glucose‐controlling effect by restorative activation of the damaged metabolic tissues by over‐nutrition. The improvement of overall metabolic dysfunction by controlling Cxxc5 function either by the CXXC5‐Dvl PPI inhibitor or by *Cxxc5* knockout could be achieved by the restorative inductions of multiple Wnt/β‐catenin pathway target genes involved in adult stem cell activation, especially those related to pathophysiological roles in metabolic diseases.^7^, ^26^, ^27^

The long‐lasting glucose‐controlling effect of KY19334 accompanying systemic metabolic improvement could be achieved by remodelling of adipose tissues by recovery of the suppressed Wnt/β‐catenin signalling and subsequently inhibits of the metabolic target genes involved in adipogenesis, lipogenesis and inflammation in matured adipocytes.

The therapeutic effects of KY19334 were also attributed to enhanced energy expenditure, which is consistent with the substantial upregulation of genes enhancing thermogenesis and browning of WAT.^28^ Additionally, restoration of functional pancreatic β‐cells through Wnt/β‐catenin signalling activation^29^ by the long‐term treatment with KY19334 also resulted in sustained blood glucose control. Differently, the prolonged glucose‐controlling effect of KY19334 did not occur with sitagliptin which is a peripheral tissue‐targeting drug.

The pathological significance of the blockade of CXXC5‐Dvl PPI was indicated by the high overexpression of CXXC5 in obese patients with diabetes and its inverse correlation with the major metabolic genes directly subjected to regulation by Wnt/β‐catenin signalling, such as *GLP1*, *TCL7L2*, *WISP1*, *c‐MYC*, *CCND1*, and *PPARδ *.^27^, ^30^, ^31^ Blockade of the CXXC5‐Dvl PPI as a strategy for the treatment of metabolic diseases was further supported by the similar physiological improvement of metabolic abnormalities in both *Cxxc5^−/−^* mice and KY19334‐treated *Cxxc5^+/+^* mice fed an HFD. Moreover, the significance of CXXC5 as a target for metabolic diseases was indicated by the absence of phenotypes of metabolic abnormalities resulting from *Cxxc5* loss.

Although previous studies supporting the role of GSK3β inhibitors in metabolic improvements through the Wnt/β‐catenin signalling activation,^32^ the GSK3β inhibitory effects could be attenuated by induction of the negative feedback regulator of CXXC5.^33^ Thus, inhibition of GSK3β activity with CXXC5‐Dvl PPI by KY19334^16^ could result in optimal activation of Wnt/β‐catenin signalling and provide subsequent therapeutic effects. The prolong activation of the Wnt/β‐catenin signalling by release of the negative feedback mechanism by CXXC5 function, differently with the direct activation by inhibition of the GSK3β alone, could offer an effective approach for long‐lasting glucose‐controlling effects, achieving remodelling of adipocytes and regeneration of pancreatic β‐cells.

Differently with the mice fed an HFD, the mice fed a NCD did not show any significant apparent abnormalities in organs and tissues, including visceral WAT, scWAT and the liver (Figure S10), suggesting safety profiles and the specificity of targeting CXXC5‐Dvl PPI for the development of drugs for metabolic diseases. Additionally, we suggest CXXC5 as a new biomarker for metabolic diseases, and its overexpression may be a major driver in the pathogenesis of multiple obesity‐related metabolic diseases.

## 5 CONCLUSIONS

Our current reports that CXXC5, a negative regulator of the Wnt/β‐catenin pathway functioning via Dvl binding, is highly expressed in visceral adipose tissues of obese‐diabetes patients. Meanwhile, *Cxxc5^−/−^* mice fed a high‐fat diet resist to systemic metabolic dysregulation. Administration of KY19334, a small molecule inhibiting the CXXC5‐Dvl interaction, results in long‐lasting glycaemic controlling effect and it is correlated with adult tissue remodelling in adaptive energy homeostasis. Overall, inhibition of CXXC5 function by small molecule‐mediated interference Dvl binding is a potential therapeutic approach for the treatment of metabolic diseases.

## CONFLICT OF INTEREST

The authors declare no competing interests exist.

## Supporting information

Supporting Information

## References

[1] Chaudhury A , Duvoor C , Dendi VSR , et al. Clinical review of antidiabetic drugs: implications for type 2 diabetes mellitus management. Front Endocrinol (Lausanne). 2017;8:6. 28167928 10.3389/fendo.2017.00006 PMC5256065

[2] DeFronzo RA , Triplitt CL , Abdul‐Ghani M , Cersosimo E . Novel agents for the treatment of type 2 diabetes. Diabetes Spectr. 2014;27:100‐112. 26246766 10.2337/diaspect.27.2.100 PMC4522879

[3] Marin‐Penalver JJ , Martin‐Timon I , Sevillano‐Collantes C , Del Canizo‐Gomez FJ . Update on the treatment of type 2 diabetes mellitus. World J Diabetes. 2016;7:354‐395. 27660695 10.4239/wjd.v7.i17.354 PMC5027002

[4] Bastakoty D , Young PP . Wnt/beta‐catenin pathway in tissue injury: roles in pathology and therapeutic opportunities for regeneration. FASEB J. 2016;30:3271‐3284. 27335371 10.1096/fj.201600502R PMC5024694

[5] Janda CY , Dang LT , You C , et al. Surrogate Wnt agonists that phenocopy canonical Wnt and beta‐catenin signalling. Nature. 2017;545:234‐237. 28467818 10.1038/nature22306 PMC5815871

[6] Wang L , Wang Y , Zhang C , et al. Inhibiting glycogen synthase kinase 3 reverses obesity‐induced white adipose tissue inflammation by regulating apoptosis inhibitor of macrophage/CD5L‐mediated macrophage migration. Arterioscler Thromb Vasc Biol. 2018;38:2103‐2116. 30026270 10.1161/ATVBAHA.118.311363

[7] Sethi JK , Vidal‐Puig A . Wnt signalling and the control of cellular metabolism. Biochem J. 2010;427:1‐17. 20226003 10.1042/BJ20091866 PMC4301310

[8] Yao DD , Yang L , Wang Y , et al. Geniposide promotes beta‐cell regeneration and survival through regulating beta‐catenin/TCF7L2 pathway. Cell Death Dis. 2015;6:e1746. 25950476 10.1038/cddis.2015.107 PMC4669687

[9] Murahovschi V , Pivovarova O , Ilkavets I , et al. WISP1 is a novel adipokine linked to inflammation in obesity. Diabetes. 2015;64:856‐866. 25281430 10.2337/db14-0444

[10] Ali H , Zmuda JM , Cvejkus RK , et al. Wnt pathway inhibitor DKK1: a potential novel biomarker for adiposity. J Endocr Soc. 2019;3:488‐495. 30746507 10.1210/js.2018-00325 PMC6364619

[11] Pontremoli M , Brioschi M , Baetta R , Ghilardi S , Banfi C . Identification of DKK‐1 as a novel mediator of statin effects in human endothelial cells. Sci Rep. 2018;8:16671. 30420710 10.1038/s41598-018-35119-7 PMC6232108

[12] Macdougall CE , Wood EG , Loschko J , et al. Visceral adipose tissue immune homeostasis is regulated by the crosstalk between adipocytes and dendritic cell subsets. Cell Metab. 2018;27:588‐601.e4. 29514067 10.1016/j.cmet.2018.02.007 PMC5846800

[13] Cohen P , Goedert M . GSK3 inhibitors: development and therapeutic potential. Nat Rev Drug Discov. 2004;3:479‐487. 15173837 10.1038/nrd1415

[14] Andersson T , Södersten E , Duckworth JK , et al. CXXC5 is a novel BMP4‐regulated modulator of Wnt signaling in neural stem cells. J Biol Chem. 2009;284:3672‐3681. 19001364 10.1074/jbc.M808119200

[15] Kim MS , Yoon SK , Bollig F , et al. A novel Wilms tumor 1 (WT1) target gene negatively regulates the WNT signaling pathway. J Biol Chem. 2010;285:14585‐14593. 20220130 10.1074/jbc.M109.094334 PMC2863207

[16] Choi S , Kim H‐Y , Cha P‐H , et al. CXXC5 mediates growth plate senescence and is a target for enhancement of longitudinal bone growth. Life Sci Alliance. 2019;2:e201800254. 30971423 10.26508/lsa.201800254 PMC6458850

[17] Kim H‐Y , Yoon J‐Y , Yun J‐H , et al. CXXC5 is a negative‐feedback regulator of the Wnt/beta‐catenin pathway involved in osteoblast differentiation. Cell Death Differ. 2015;22:912‐920. 25633194 10.1038/cdd.2014.238 PMC4423189

[18] Seo SH , Kim E , Joo Y , et al. A mixed micellar formulation for the transdermal delivery of an indirubin analog. Pharmaceutics. 2020;12:175. 10.3390/pharmaceutics12020175 PMC7076637 32093032

[19] Kim J , Moon J , Park C‐H , et al. NT‐PGC‐1alpha deficiency attenuates high‐fat diet‐induced obesity by modulating food intake, fecal fat excretion and intestinal fat absorption. Sci Rep. 2021;11:1323. 33446719 10.1038/s41598-020-79823-9 PMC7809341

[20] Salvalaggio PR , Deng S , Ariyan CE , et al. Islet filtration: a simple and rapid new purification procedure that avoids ficoll and improves islet mass and function. Transplantation. 2002;74:877‐879. 12364870 10.1097/00007890-200209270-00023

[21] Ghorpade DS , Ozcan L , Zheng Z , et al. Hepatocyte‐secreted DPP4 in obesity promotes adipose inflammation and insulin resistance. Nature. 2018;555:673‐677. 29562231 10.1038/nature26138 PMC6021131

[22] Monteiro R , Azevedo I . Chronic inflammation in obesity and the metabolic syndrome. Mediators Inflamm. 2010;2010:289645. 20706689 10.1155/2010/289645 PMC2913796

[23] Schenk S , Saberi M , Olefsky JM . Insulin sensitivity: modulation by nutrients and inflammation. J Clin Invest. 2008;118:2992‐3002. 18769626 10.1172/JCI34260 PMC2522344

[24] Prentki M , Nolan CJ . Islet beta cell failure in type 2 diabetes. J Clin Invest. 2006;116:1802‐1812. 16823478 10.1172/JCI29103 PMC1483155

[25] Ferrand N , Béreziat V , Moldes M , Zaoui M , Larsen AK , Sabbah M . WISP1/CCN4 inhibits adipocyte differentiation through repression of PPARgamma activity. Sci Rep. 2017;7:1749. 28496206 10.1038/s41598-017-01866-2 PMC5431985

[26] Boj SF , van Es JH , Huch M , et al. Diabetes risk gene and Wnt effector Tcf7l2/TCF4 controls hepatic response to perinatal and adult metabolic demand. Cell. 2012;151:1595‐1607. 23260145 10.1016/j.cell.2012.10.053

[27] Choi OM , Cho Y‐H , Choi S , et al. The small molecule indirubin‐3'‐oxime activates Wnt/beta‐catenin signaling and inhibits adipocyte differentiation and obesity. Int J Obes (Lond). 2014;38:1044‐1052. 24232498 10.1038/ijo.2013.209 PMC4125748

[28] Zhang G , Sun Q , Liu C . Influencing factors of thermogenic adipose tissue activity. Front Physiol. 2016;7:29. 26903879 10.3389/fphys.2016.00029 PMC4742553

[29] Ali A , Ali A , Ahmad W , et al. Deciphering the role of WNT signaling in metabolic syndrome‐linked Alzheimer's disease. Mol Neurobiol. 2020;57:302‐314. 31325024 10.1007/s12035-019-01700-y

[30] Bennett CN , Ross SE , Longo KA , et al. Regulation of Wnt signaling during adipogenesis. J Biol Chem. 2002;277:30998‐31004. 12055200 10.1074/jbc.M204527200

[31] Ross SE , Hemati N , Longo KA , et al. Inhibition of adipogenesis by Wnt signaling. Science. 2000;289:950‐953. 10937998 10.1126/science.289.5481.950

[32] Maqbool M , Hoda N . GSK3 Inhibitors in the therapeutic development of diabetes, cancer and neurodegeneration: past, present and future. Curr Pharm Des. 2017;23:4332‐4350. 28714403 10.2174/1381612823666170714141450

[33] Kim HY , Choi S , Yoon J‐H , et al. Small molecule inhibitors of the dishevelled‐CXXC5 interaction are new drug candidates for bone anabolic osteoporosis therapy. EMBO Mol Med. 2016;8:375‐387. 26941261 10.15252/emmm.201505714 PMC4818757

