# Supplementary material for: Inhibition of CXXC5 function reverses obesity‐related metabolic diseases
Source: Clin Transl Med. 2022 Apr 5;12(4):e742. doi: 10.1002/ctm2.742 (PMC8982507; doi:10.1002/ctm2.742)
Supplement: Supplementary file 1 — Supporting Information [file CTM2-12-e742-s001.pdf]

1 **Supplementary Information**

Fig. S1

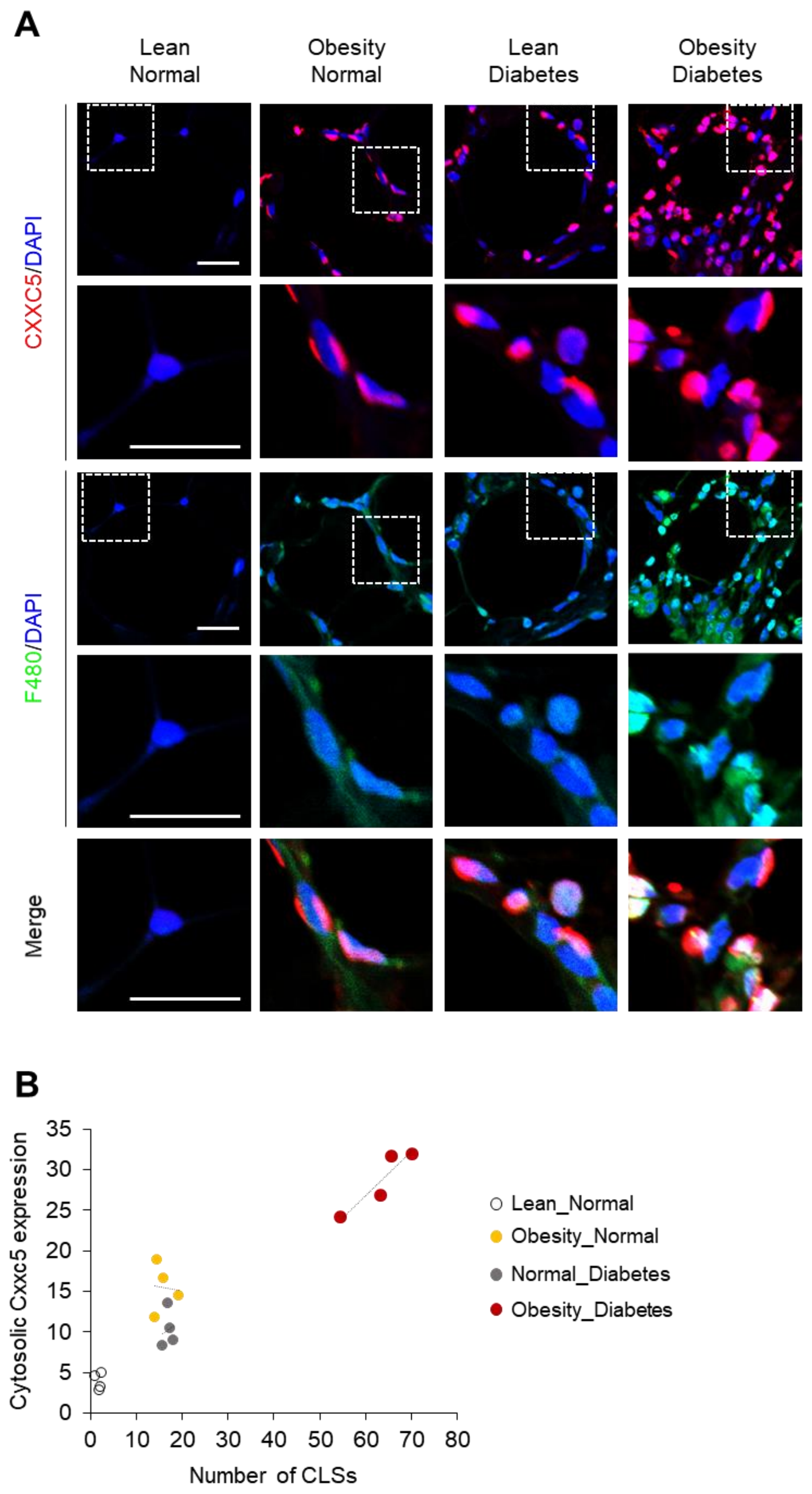

2

Figure S1. CXXC5 is expressed in F4/80 positive CLSs. Visceral adipose tissues from human subjects that were lean, obese, diabetic, and obese-diabetic (n = 4 per group). **(A)** Representative IHC images of CXXC5 and F4/80 in visceral adipose tissue. **(B)** The correlation of cytosolic CXXC5 expression with the number of CLSs. Scale bars = 100 μm.

Fig. S2

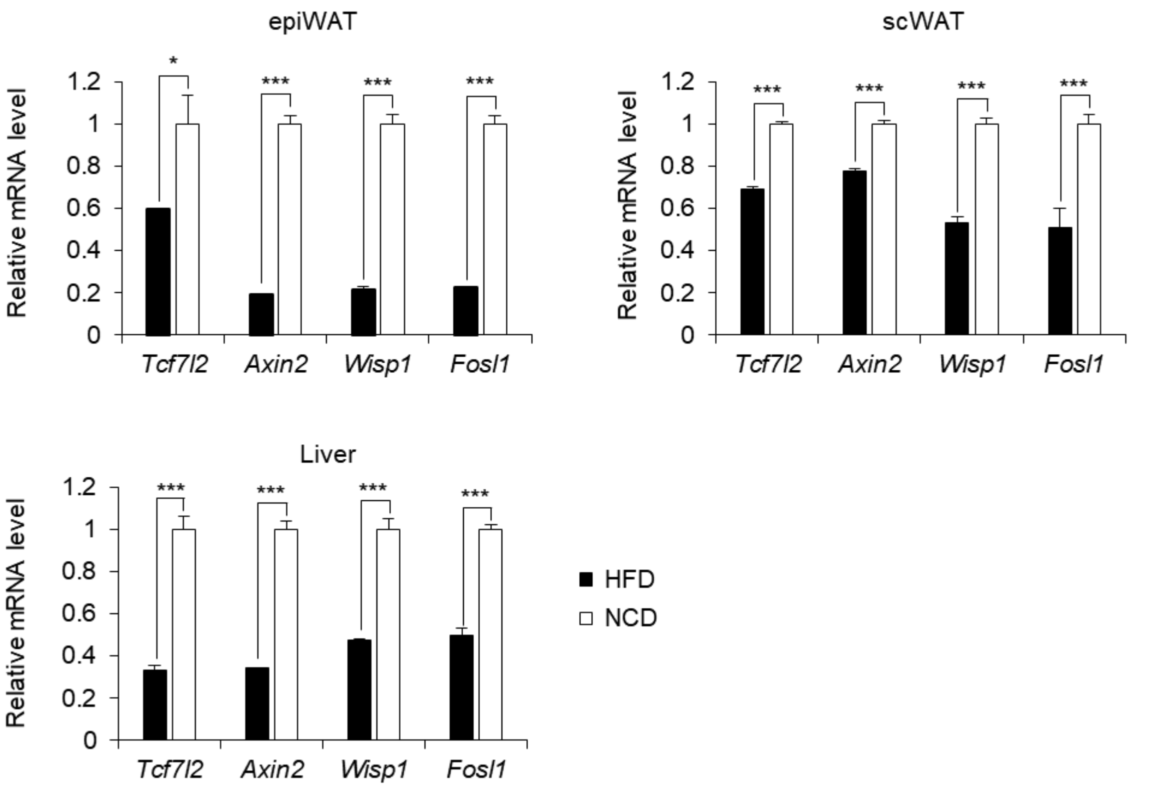

Figure S2. Analyses of the expression levels of Wnt/β-catenin signaling target genes in insulin sensitive tissues. *Cxxc5*$^{+/+}$ mice fed HFD or NCD for 8 weeks (n = 6 per group). Relative mRNA expression levels of Wnt/β-catenin signaling target genes (*Tcf7l2*, *Axin2*, *Wisp1*, and *Fosl1*) in epiWAT, scWAT, and liver. Expression levels of mRNA were normalized by HFD-fed group. All data are presented as the mean ± SD. $*P < 0.05$, $**P < 0.01$, $***P < 0.001$ determined by Student's t-test.

Fig. S3

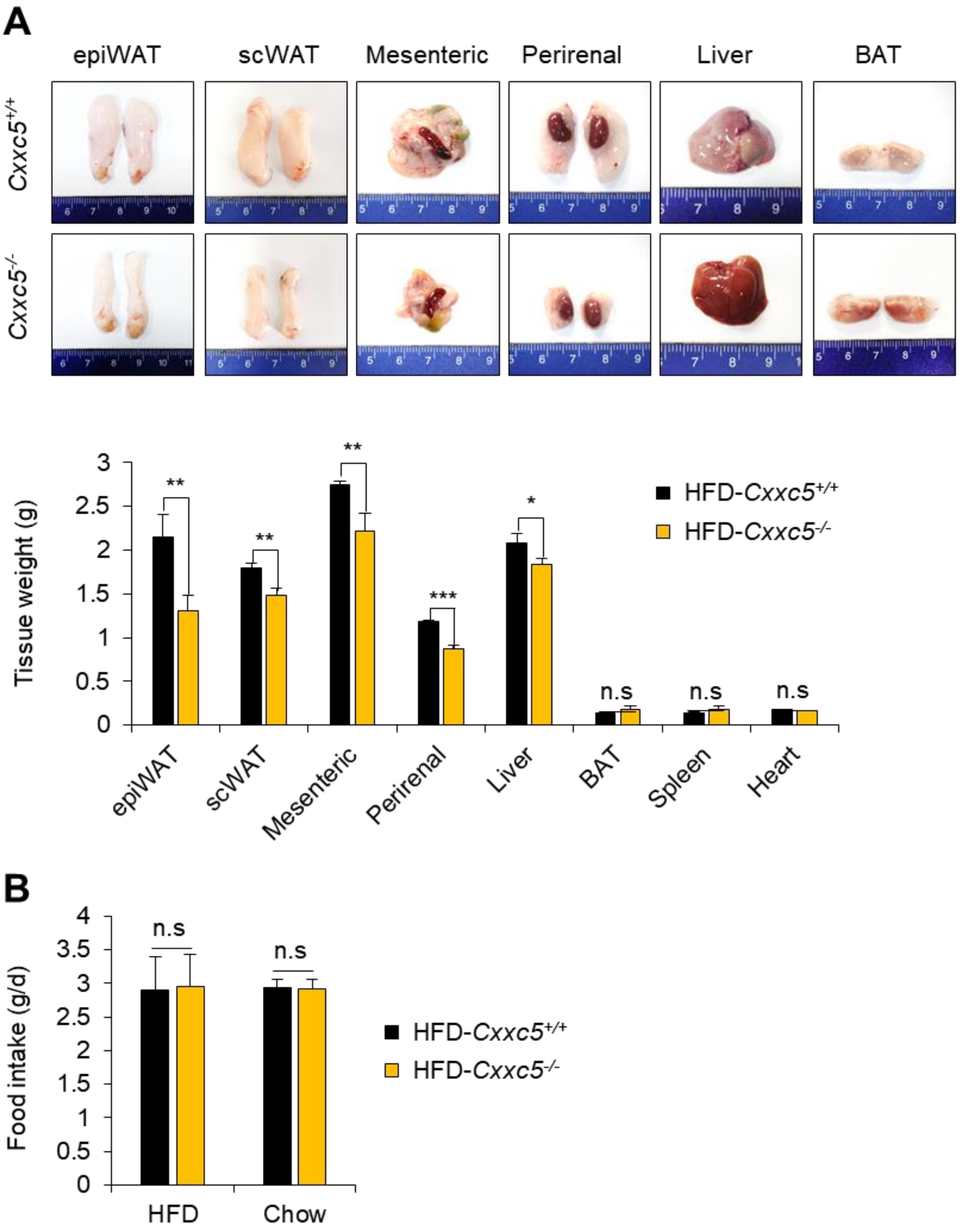

Figure S3. Ablation of *Cxxc5* resists obesity without differences in food intake. *Cxxc5*$^{+/+}$ and *Cxxc5*$^{-/-}$ mice fed HFD for 8 weeks (n = 9–13 per group). **(A)** Representative photographs (upper panel) and wet weight of epiWAT, scWAT, mesenteric, perirenal, liver, BAT, spleen, and heart (lower panel). **(B)** Daily food intake during all study weeks. All data are presented as the mean ± SD. *$P < 0.05$, **$P < 0.01$, ***$P < 0.001$ determined by Student's t-test.

Fig. S4

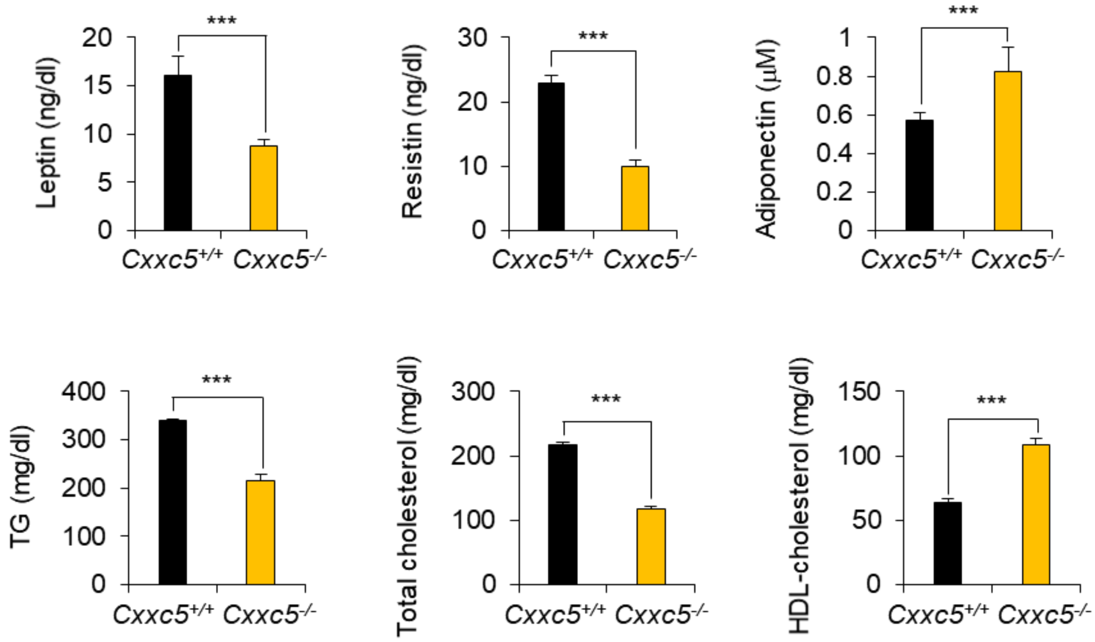

Figure S4. Ablation of *Cxxc5* improves metabolic parameters. *Cxxc5*$^{+/+}$ and *Cxxc5*$^{-/-}$ mice were fed HFD for 8 weeks (n = 9–13 per group). Plasma concentration or relative levels of leptin, resistin, adiponectin, TGs, total cholesterol, and HDL-cholesterol after overnight fasting. All data are presented as the mean ± SD. ***$P < 0.001$ determined by Student’s *t*-test.

Fig. S5

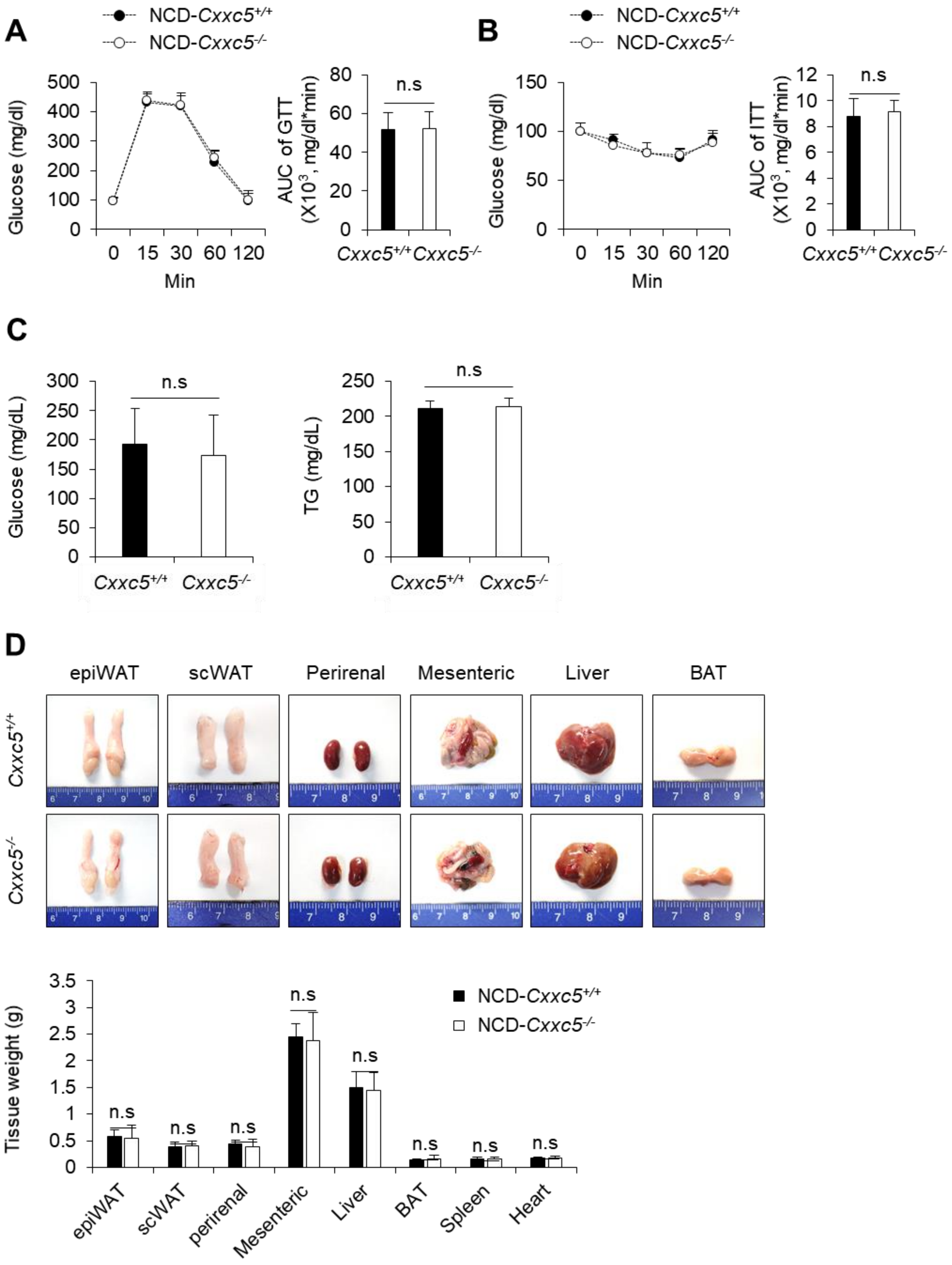

A
NCD-Cxxc5+/+
NCD-Cxxc5-/-
Glucose (mg/dl)
500
400
300
200
100
0
0 15 30 60 120
Min
AUC of GTT (X10³, mg/dl*min)
80
60
40
20
0
n.s
Cxxc5+/+ Cxxc5-/-
B
NCD-Cxxc5+/+
NCD-Cxxc5-/-
Glucose (mg/dl)
150
100
50
0
0 15 30 60 120
Min
AUC of ITT (X10³, mg/dl*min)
12
10
8
6
4
2
0
n.s
Cxxc5+/+ Cxxc5-/-
C
Glucose (mg/dL)
300
250
200
150
100
50
0
n.s
Cxxc5+/+ Cxxc5-/-
TG (mg/dL)
250
200
150
100
50
0
n.s
Cxxc5+/+ Cxxc5-/-
D
epiWAT
scWAT
Perirenal
Mesenteric
Liver
BAT
Cxxc5+/+
Cxxc5-/-
Tissue weight (g)
3.5
3
2.5
2
1.5
1
0.5
0
n.s
NCD-Cxxc5+/+
NCD-Cxxc5-/-
epiWAT
scWAT
perirenal
Mesenteric
Liver
BAT
Spleen
Heart

Figure S5. Ablation of *Cxxc5* has no metabolic effects on mice fed NCD. *Cxxc5*$^{+/+}$ and *Cxxc5*$^{-/-}$ mice were fed NCD for 8 weeks (n = 9–12 per group). **(A)** Glucose tolerance test and AUC. **(B)** Insulin tolerance test and AUC. **(C)** Plasma concentration of glucose and TGs. **(D)** Representative photographs (upper panel) and wet weight of epiWAT, scWAT, perirenal, mesenteric, liver, BAT, spleen, and heart (lower panel). All data are presented as the mean ± SD. Statistical analysis was determined by Student's *t*-test.

Fig. S6

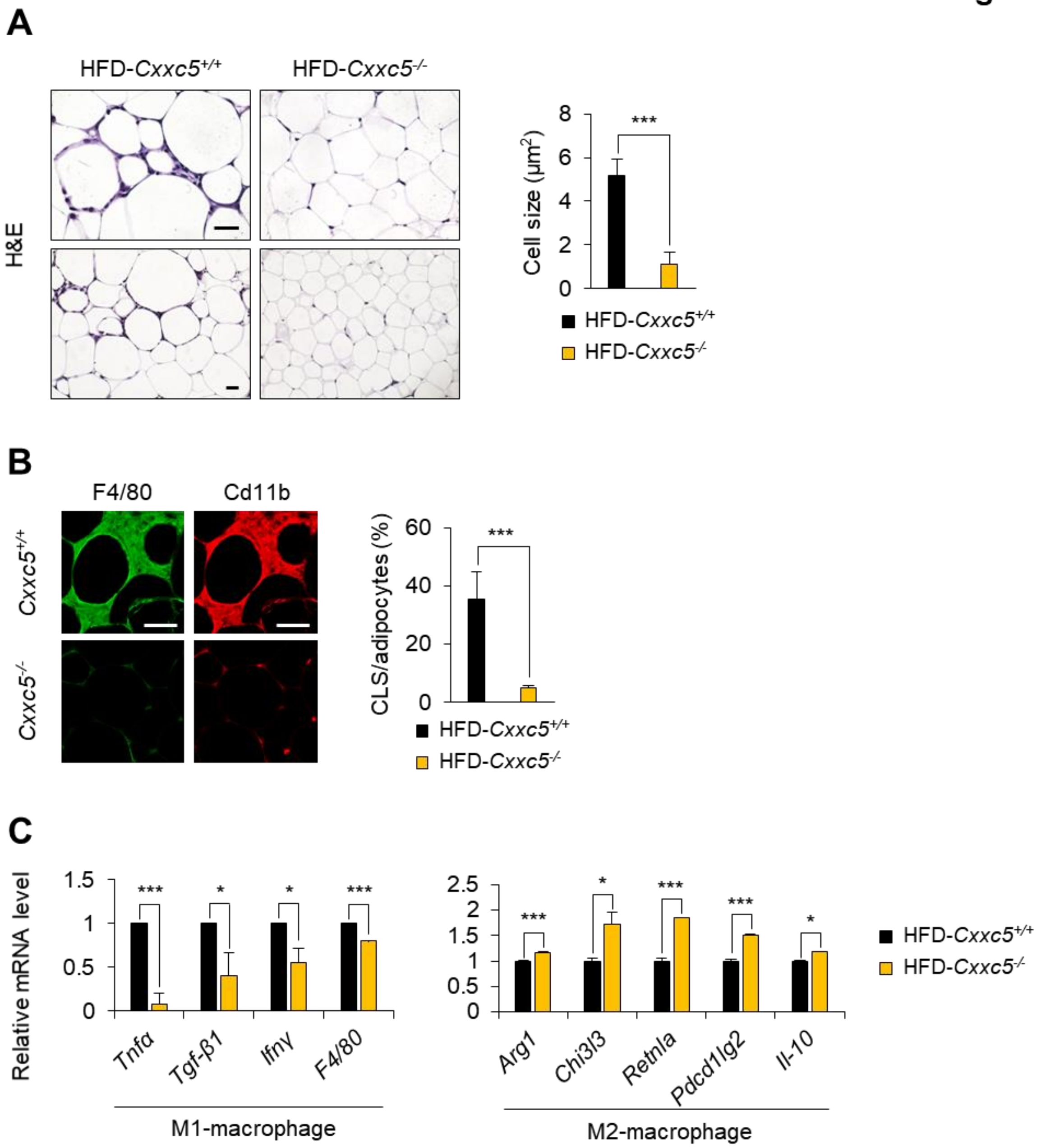

Figure S6. Ablation of *Cxxc5* resists hypertrophy of adipose tissue with modulation of M1 and M2 macrophage markers. epiWAT from *Cxxc5*$^{+/+}$ and *Cxxc5*$^{-/-}$ mice fed HFD for 8 weeks (n = 9–13 per group). **(A)** Representative images of H&E staining (left panel). Quantitative analyses of adipocyte cell size (right panel). **(B)** Representative IHC images for F4/80 and Cd11b (left panel) and the percentage of crown-like structures (CLSs) per adipocyte on

histological sections (right panel). **(C)** Relative expression levels of marker genes for M1 and M2 macrophages. Expression levels of mRNA were normalized by HFD-fed *Cxxc5*$^{+/+}$ mice group. All data are presented as the mean ± SD. *P < 0.05, **P < 0.01, ***P < 0.001 determined by Student's t-test.

Fig. S7

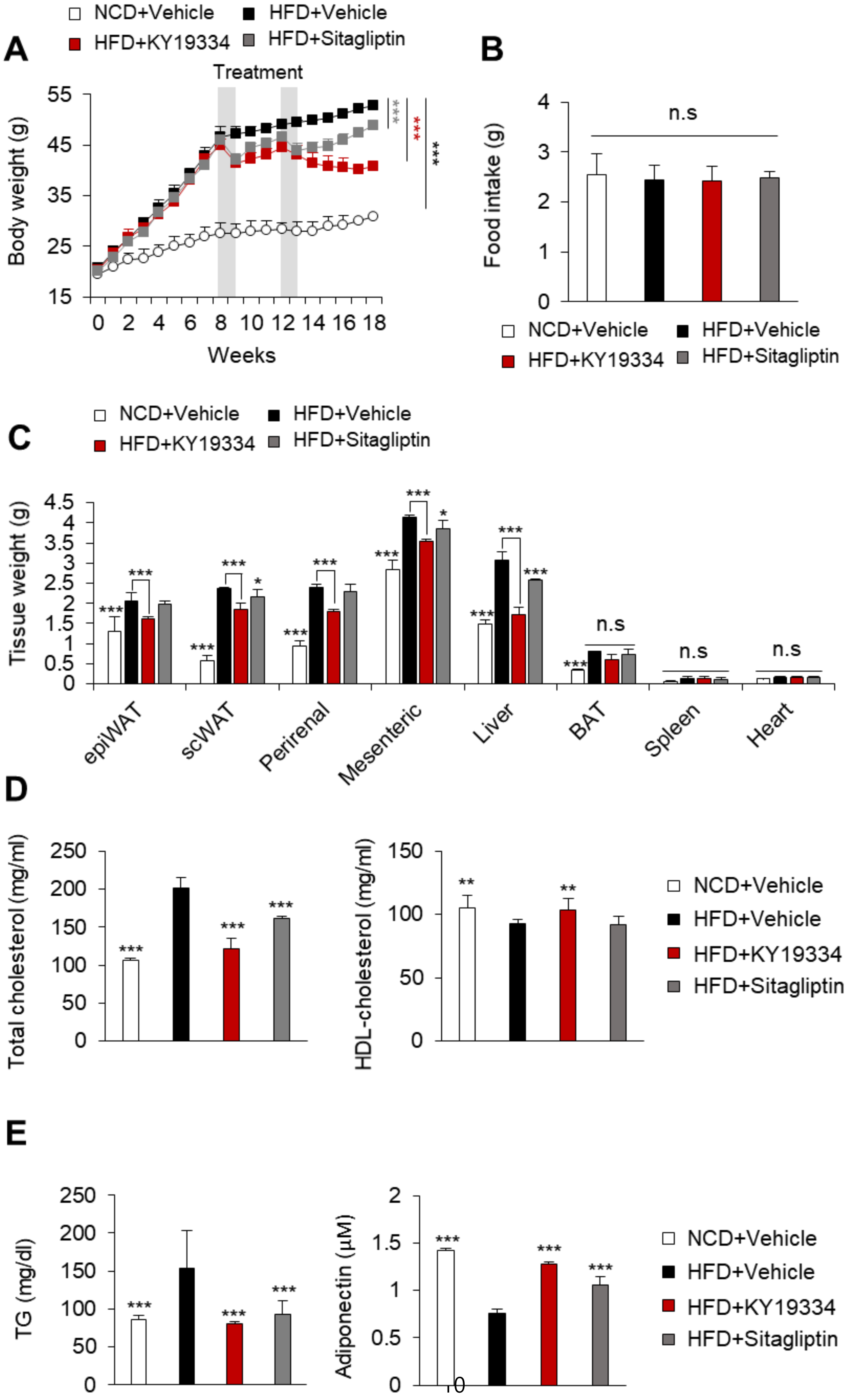
A
NCD+Vehicle
HFD+Vehicle
HFD+KY19334
HFD+Sitagliptin
Treatment
Body weight (g)
Weeks
B
Food intake (g)
n.s
C
Tissue weight (g)
epiWAT
scWAT
Perirenal
Mesenteric
Liver
BAT
Spleen
Heart
D
Total cholesterol (mg/ml)
HDL-cholesterol (mg/ml)
E
TG (mg/dl)
Adiponectin (μM)

Figure S7. Relative effectiveness of KY19334 and sitagliptin on diet-induced obesity. C57BL/6 mice fed NCD or HFD for 18 weeks were p.o. administered KY19334 (25 mg/kg/d), sitagliptin (50 mg/kg/d) for 5 days on weeks 8 and 12 (*n* = 10 per group). **(A)** Body weight changes. **(B)** Daily food intake during all study wks. **(C)** Wet weight of epiWAT, scWAT, perirenal, mesenteric, liver, BAT, spleen, and heart. **(D, E)** Plasma concentration after overnight fasting. Total cholesterol and HDL-cholesterol **(D)**, TGs, and adiponectin **(E)**. All data are presented as the mean ± SD. *$P < 0.05$, ***$P < 0.001$ determined by Student's *t*-test.

Fig. S8

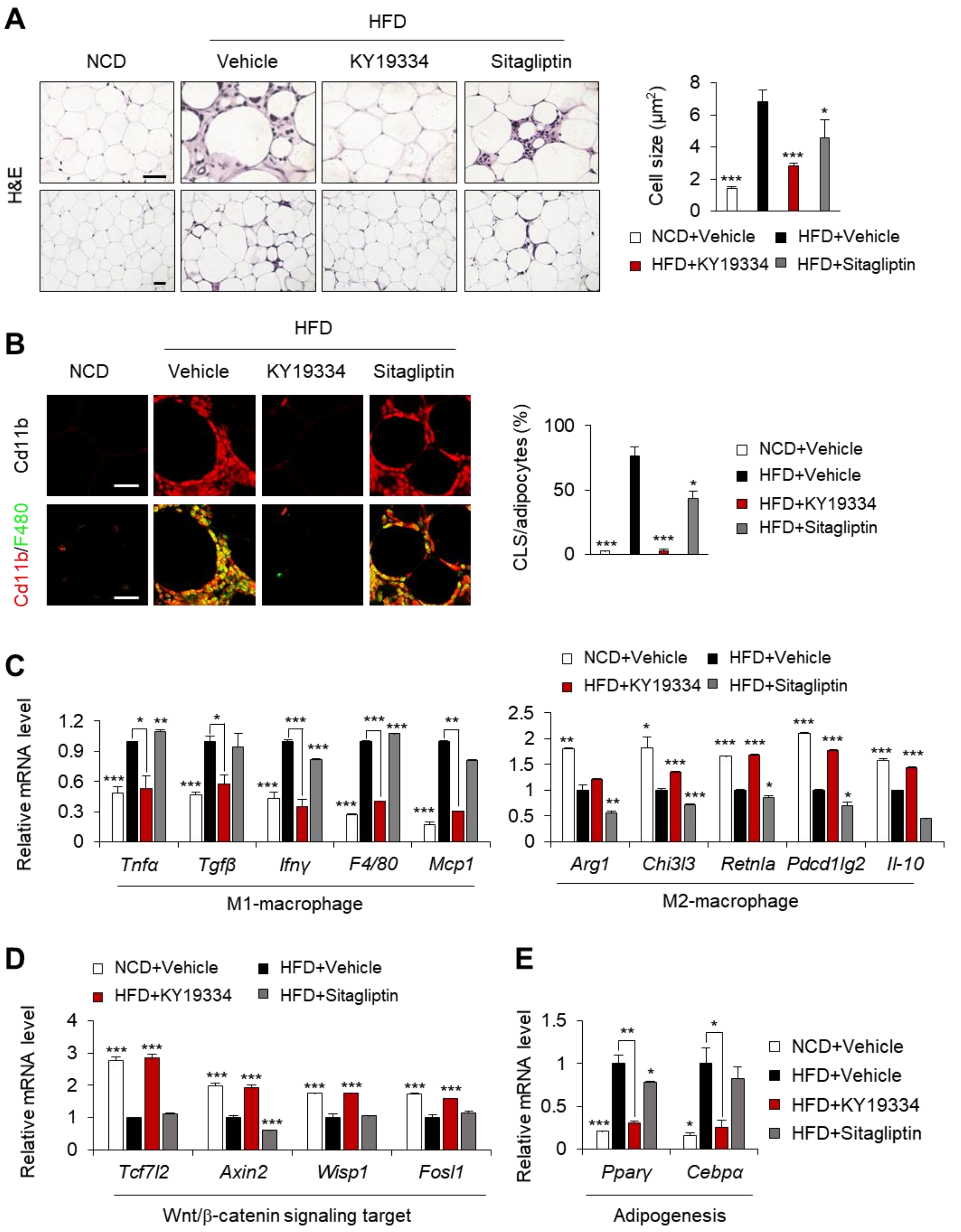
A
HFD
NCD
Vehicle
KY19334
Sitagliptin
H&E
Cell size (μm²)
NCD+Vehicle
HFD+Vehicle
HFD+KY19334
HFD+Sitagliptin
B
Cd11b
Cd11b/F480
CLS/adipocytes (%)
C
Relative mRNA level
Tnfα
Tgfβ
Ifnγ
F4/80
Mcp1
M1-macrophage
Arg1
Chi3l3
Retnla
Pdcd1lg2
Il-10
M2-macrophage
D
Tcf7l2
Axin2
Wisp1
Fosl1
Wnt/β-catenin signaling target
E
Pparγ
Cebpα
Adipogenesis

Figure S8. KY19334 induces adipose tissue remodeling involving improvement inflammation and adipogenesis. C57BL/6 mice fed NCD or HFD for 18 weeks were p.o. administered KY19334 (25 mg/kg/d) or sitagliptin (50 mg/kg/d) for 5 days on weeks 8 and 12 (*n* = 10 per group). **(A)** Representative images of H&E staining of epiWAT (left panel) and quantitative analyses of adipocyte cell size of epiWAT (right panel). **(B)** Representative IHC images (*n* = 5 independent experiments) for F4/80 and Cd11b (left panel) and the percentage of crown-like structures (CLSs) per adipocytes on histological sections (right panel). **(C)** Flow cytometry analysis of the expression of F4/80 and Cd11b and percentage of F4/80^{+}Cd11b^{+} cells are shown. **(D-F)** Relative mRNA expression of M1 and M2 macrophage markers **(D)**, Wnt/β-catenin signaling target **(E)**, and adipogenesis **(F)** genes. Expression levels of mRNA were normalized by vehicle-treated HFD mice group. Scale bars = 100 μm. All data are presented as the mean ± SD. *$P < 0.05$, **$P < 0.01$, ***$P < 0.001$ determined by Student's *t*-test.

Fig. S9

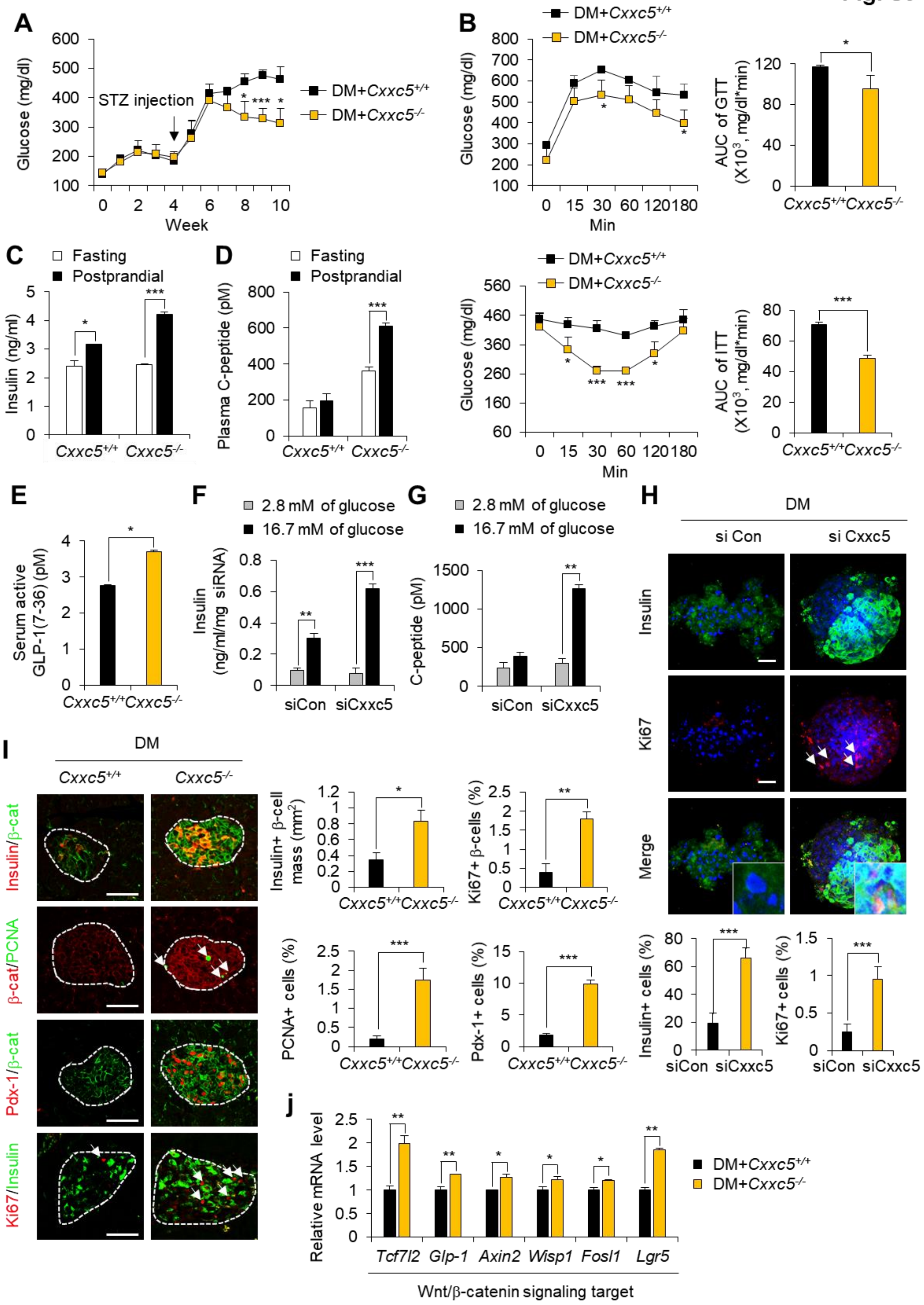
A
Glucose (mg/dl)
STZ injection
Week
DM+Cxxc5+/+
DM+Cxxc5-/-
B
Glucose (mg/dl)
Min
AUC of GTT (X10³, mg/dl*min)
Cxxc5+/+ Cxxc5-/-
C
Fasting
Postprandial
Insulin (ng/ml)
D
Plasma C-peptide (pM)
Glucose (mg/dl)
AUC of ITT (X10³, mg/dl*min)
E
Serum active GLP-1(7-36) (pM)
F
2.8 mM of glucose
16.7 mM of glucose
Insulin (ng/ml/mg siRNA)
siCon siCxxc5
G
C-peptide (pM)
H
DM
si Con
si Cxxc5
Insulin
Ki67
Merge
I
DM
Insulin/β-cat
β-cat/PCNA
Pdx-1/β-cat
Ki67/Insulin
Insulin+ β-cell mass (mm²)
Ki67+ β-cells (%)
PCNA+ cells (%)
Pdx-1+ cells (%)
Insulin+ cells (%)
Ki67+ cells (%)
j
Relative mRNA level
Tcf7l2 Glp-1 Axin2 Wisp1 Fosl1 Lgr5
Wnt/β-catenin signaling target

160

Figure S9. Ablation of *Cxxc5* preserves β-cell mass and functions in HFD-fed and STZ-induced diabetes mellitus (DM) mice. *Cxxc5*$^{+/+}$ and *Cxxc5*$^{-/-}$ mice fed HFD for 4 weeks followed by injection with STZ (50 mg/kg/d) for 1 week (n = 6 per group). **(A)** Non-fasting blood glucose levels. **(B)** Glucose tolerance (upper panel) and insulin tolerance tests (lower panel) and AUC. **(C-E)** Plasma concentration of insulin **(C)**, C-peptide **(D)**, and serum active GLP-1 levels **(E)**. **(F-H)** Isolated islets from the pancreas of DM-induced *Cxxc5*$^{+/+}$ and *Cxxc5*$^{-/-}$ mice. For transient transfection, islets were transfected with 2μg of siRNA using lipofectamine in Opti-MEM. The concentration of secreted insulin **(F)** and c-peptide **(G)** from islets in response to different concentrations was measured after incubation for 1 h with either low (2.8 mM) or high (16.7 mM) glucose in KRBH buffer. **(H)** Representative images of immunofluorescent staining for insulin and Ki67 (upper panel). Quantitative analyses of insulin and Ki67 positive cells in the islets (lower panel). **(I)** Representative images of immunofluorescent staining for β-catenin, insulin, PCNA, Pdx-1, and Ki67 (left panel). Quantitative analyses of insulin-positive β-cell mass, insulin content, PCNA, Pdx-1, and Ki67 positive cells in the pancreatic tissues (right panel). **(J)** Relative expression levels of mRNAs for the Wnt/β-catenin signaling target genes. Expression levels of mRNA were normalized by DM-induced *Cxxc5*$^{+/+}$ mice group. Scale bars = 100 μm. All data are presented as the mean ± SD. *$P < 0.05$, ***$P < 0.001$ determined by Student's *t*- test. DM: Diabetes mellitus.

Fig. S10

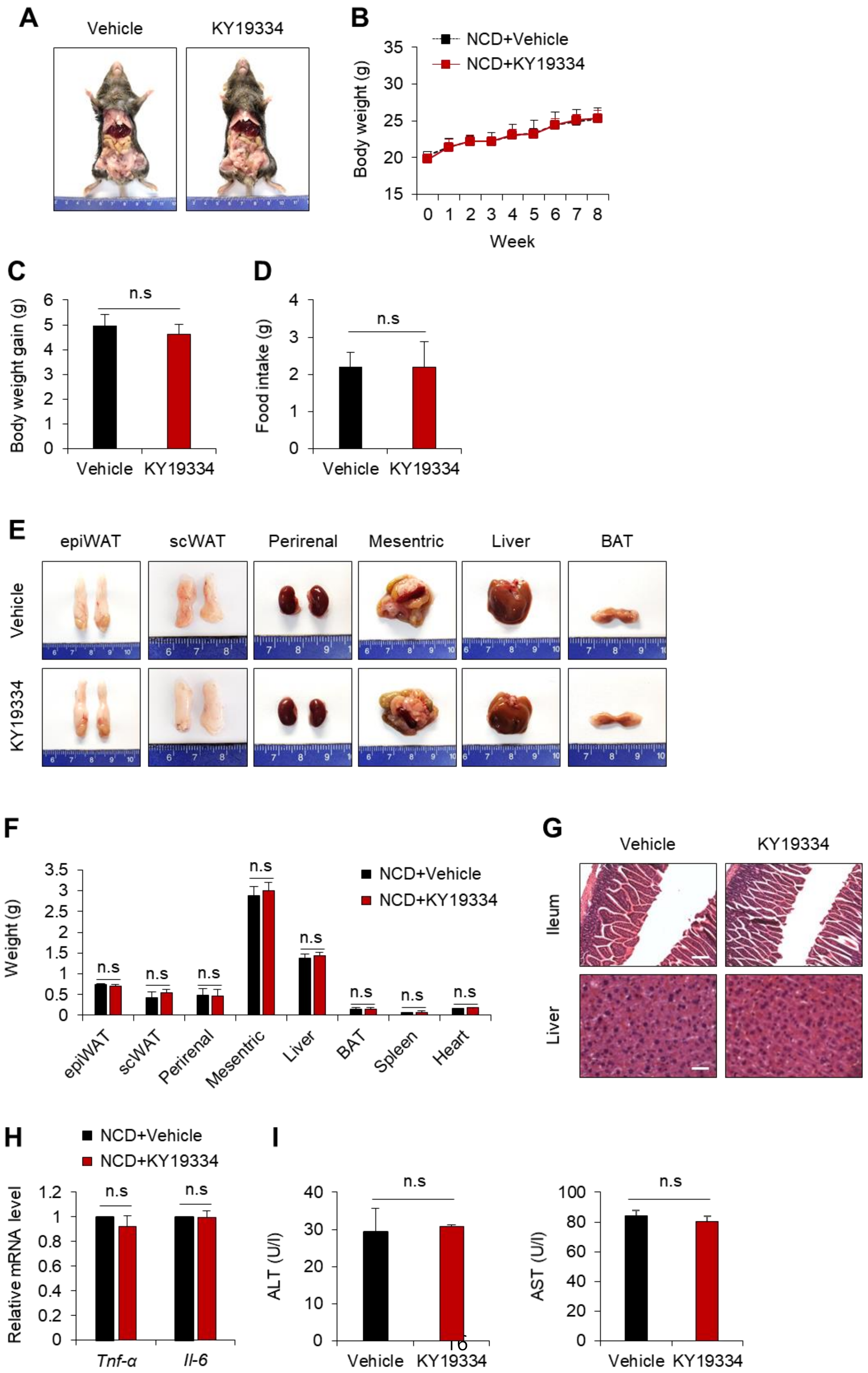

A
Vehicle
KY19334
B
NCD+Vehicle
NCD+KY19334
Body weight (g)
35
30
25
20
15
0 1 2 3 4 5 6 7 8
Week
C
Body weight gain (g)
n.s
Vehicle KY19334
D
Food intake (g)
n.s
Vehicle KY19334
E
epiWAT
scWAT
Perirenal
Mesentric
Liver
BAT
Vehicle
KY19334
F
Weight (g)
n.s
NCD+Vehicle
NCD+KY19334
epiWAT
scWAT
Perirenal
Mesentric
Liver
BAT
Spleen
Heart
G
Vehicle
KY19334
Ileum
Liver
H
NCD+Vehicle
NCD+KY19334
Relative mRNA level
n.s
Tnf-α
Il-6
I
ALT (U/l)
n.s
Vehicle KY19334
AST (U/l)
n.s
Vehicle KY19334

184

Figure S10. KY19334 treatment does not show any metabolic effects in mice fed NCD. NCD-fed C57BL/6 mice were p.o. administered KY19334 (25 mg/kg/d) for 8 weeks (n = 10 per group). **(A)** Representative photographs of vehicle- or KY19334-treated mice. **(B)** Body weight changes. **(C)** Body weight gain. **(D)** Food intake. **(E)** Representative photographs of fat pads (epiWAT, BAT), mesenteric, perirenal, and liver. **(F)** Wet weight of epiWAT, mesenteric, perirenal, liver, BAT, and heart. **(G)** Representative images (three total images per group) of H&E staining of ileum and liver tissue. **(H)** Relative expression levels of *Tnf-α* and *Il-6*. Expression levels of mRNA were normalized by NCD-fed vehicle group. **(I)** Plasma concentrations of ALT and AST. Scale bars = 100 µm. All data are presented as the mean ± SD. n.s = non significance.

Table S1. Sequences of real-time PCR primers used in this study.

Gene	Forward	Reverse
Axin2	5’-TGGAGAGTGAGCGGCAGAGC-3’	5’-TGGAGACGAGCGGGCAGA-3’
Wisp1	5’-ATCGCCCGAGGTACGCAATAGG-3’	5’-CAGCCCACCGTGCCATCAATG-3’
Fosl1	5’-AACCGGAGGAAGGAACTGAC-3’	5’-CTGCAGCCCAGATTTCTCA-3’
Cxxc5	5’-CAAGAAGAAGCGGAAACGCTGC-3’	5’-TCTCCAGAGCAGCGGAAGGCTT-3’
Tcf7l2	5’-TGTGTACCCAATCACGACAGGAG-3’	5’-GATTCCGGTCGTGTGCAGAG-3’
Tnfα	5’-CGGAGTCCGGGCAGGT-3’	5’-GCTGGGTAGAGAATGGATCA-3’
Tgfβ1	5’-TGACGTCACTGGAGTTGTACGG-3’	5’-GGTTCATGTCATGGATGGTGC-3’
Ifnγ	5’-TCAAGTGGCATAGATGTGGAAGAA-3’	5’-TGGCTCTGCAGGATTTTCATG-3’
F4/80	5’-CTTTGGCTATGGGCTTCCAGTC-3’	5’-GCAAGGAGGACAGAGTTTATCGTG-3’
Mcp1	5’-ACTGAAGCCAGCTCTCTCTTCCTC-3’	5’-TTCCTTCTTGGGGTCAGCACAGAC-3’
Arg1	5’-CTCCAAGCCAAAGTCCTTAGAG-3’	5’-GGAGCTGTCATTAGGGACATCA-3’
Chi3l3	5’-CAGGTCTGGCAATTCTTCTGAA-3’	5’-GTCTTGCTCATGTGTGTAAGTGA-3’
Retnla	5’-CCAATCCAGCTAACTATCCCTCC-3’	5’-ACCCAGTAGCAGTCATCCCA-3’
Pdcd1lg2	5’-TTGTCGGTGTGATTGGCTTC-3’	5’-AAAAGGCAGCACACAGTTGC-3’
Il-10	5’-GCTATGCTGCCTGCTCTTACT-3’	5’-CCTGCTGATCCTCATGCCA-3’
Pparδ	5’-TCCATCGTCAACAAAGACGGG-3’	5’-ACTTGGGCTCAATGATGTCAC-3’
Pparγ	5’-TGTGGGGATAAAGCATCAGGC-3’	5’-CCGGCAGTTAAGATCACACCTAT-3’
Cecpα	5’-GGTGGACAAGAACAGCAACGA-3’	5’-TGTCCAGTTCACGGCTCAGCT-3’
Srebp1	5’-GGAGCCATGGATTGCACATT-3’	5’-GGCCCGGGAAGTCACTGT-3’
Fas	5’-GCGATGAAGAGCATGGTTTAG-3’	5’-GGCTCAAGGGTTCCATGTT-3’
Scd-1	5’-CTGTACGGGATCATACTGGTTC-3’	5’-GCCGTGCCTTGTAAGTTCTG-3’
Acc	5’-CCTCCGTCAGCTCAGATACA-3’	5’-TTTACTAGGTGCAAGCCAGACA-3’
G6pc	5’-GTCGTGGCTGGAGTCTTG-3’	5’-CGGAGGCTGGCATTGTAG-3’
Pepck	5’-ATCTCCTTTGGAAGCGGATATG-3’	5’-CGCAACGCAAAGCATTTCTT-3’
Pck1	5’-GGTATTGAACTGACAGACTC-3’	5’-CCAGTTGTTGACCAAAGG-3’
Fbp1	5’-GTAACATCTACAGCCTTAATGAG-3’	5’-CCAGAGTGCGGTGAATATC-3’

Ucp1	5’-AGGCTTCCAGTACCATTAGGT-3’	5’-CTGAGTGAGGCAAAGCTGATTT-3’
Pgc-1α	5’-AGCCGTGACCACTGACAACGAG-3’	5’-GCTGCATGGTTCTGAGTGCTAAG-3’
Prdm16	5’-CCACCAGCGAGGACTTCAC-3’	5’-GGAGGACTCTCGTAGCTCGAA-3’
Elovl3	5’-TTCTCACGCGGGTTAAAAATGG-3’	5’-GAGCAACAGATAGACGACCAC-3’
Cox8b	5’-GAACCATGAAGCCAACGACT-3’	5’-GCGAAGTTCACAGTGGTTCC-3’
Cd137	5’-CCTTGCAGGTCCTTACCTTGT-3’	5’-GTTGCTTGAATATGTGGGGGA-3’
Tmem26	5’-ATGGTGCATTTCAAGAAGCC-3’	5’-GCTCACCCTCAAGTTCAAGC-3’
Tbx1	5’-CTGTGGGACGAGTTCAATCAG-3’	5’-TTGTCATCTACGGGCACAAAG
